# Various diseases and conditions are strongly associated with the next-generation epigenetic aging clock CheekAge

**DOI:** 10.1007/s11357-025-01579-9

**Published:** 2025-03-07

**Authors:** Maxim N. Shokhirev, Adiv A. Johnson

**Affiliations:** Tally Health, New York, NY USA

**Keywords:** Aging biomarker, Epigenetic clock, Aging clock, Buccal, Age-related disease, DNA methylation

## Abstract

Epigenetic aging clocks represent contemporary aging biomarkers that predict age using methylomic data. These models can be categorized as first-generation clocks that estimate chronological age or next-generation clocks that are designed to associate with health, lifestyle, and/or outcomes. Recently, we created a next-generation buccal clock called CheekAge that associates with all-cause mortality risk in older adults. To better understand our model, we collated 25 Infinium MethylationEPIC datasets in the Gene Expression Omnibus database and analyzed the ability of CheekAge and five other well-known clocks to associate with distinct health and disease signals. CheekAge outcompeted every other clock tested by significantly associating with a total of 33 different disease and health variables, including human immunodeficiency virus, major depressive disorder, psychological trauma, prediabetes, body mass index, non-alcoholic fatty liver disease, pulmonary fibrosis, exposure to the chemical endocrine disruptor PBB-153, and various cancers and tumors. Of the six clocks tested, the next-generation clocks outperformed the first-generation clocks. To better understand the underlying biology of CheekAge, we iteratively removed CpG inputs to identify DNA methylation sites that promoted or antagonized each association. Finally, we performed detailed enrichment analyses on these sites to unveil overrepresented biological processes and transcription factor targets.

**Supplementary Information:**

The online version contains supplementary material available at 10.1007/s11357-025-01579-9.

## Introduction

Aging biomarkers are metrics that provide information beyond chronological age when it comes to predicting age-related outcomes like all-cause mortality [1]. A finite number of validated aging biomarkers have been identified, including the frailty index [2], grip strength [3], cardiorespiratory fitness [4], and select epigenetic aging clocks. The latter represent computational models that predict age given DNA methylation information as inputs. These clocks are commonly trained using the supervised machine learning methods elastic net or lasso [5]. Many other methods have also been employed, such as multivariate linear regression [6], random forest [7], and deep learning [8]. The output of these models is frequently referred to as either epigenetic age or DNA methylation age and, broadly speaking, epigenetic aging clocks can be divided into first-generation and next-generation categories [9]. Early, first-generation models like the Hannum 2013 [10] and Horvath 2013 [11] clocks were trained to predict chronological age with a high accuracy. More contemporary, next-generation clocks are trained to capture health, lifestyle, and/or outcomes in addition to predicting age. The first next-generation clock created was DNAm PhenoAge [12], which is blood-based.

More recently, we developed a next-generation epigenetic aging clock using buccal Infinium MethylationEPIC data derived from a large, diverse population of more than 8000 adults. Buccal tissue was chosen because cheek swabs are painless, easy to collect, and can be performed in a home setting. Furthermore, previously published work had shown that cheek swabs are a viable tissue source for epigenetic age prediction [13, 14]. The created model, which we call CheekAge, showed robust associations with holistic lifestyle and health factors in our internally collected data [15]. Moreover, CheekAge was significantly associated with all-cause mortality risk in a longitudinal blood dataset derived from older adults and outperformed all first-generation clocks tested [16]. We additionally performed some early, exploratory analyses in publicly available external datasets, showing that CheekAge was elevated in patients with a COVID infection, progeria, malignant meningioma, and a high body mass index. We also found that CheekAge was increased in childhood cancer survivors treated with radiation therapy [15]. The key take-away from this work was that, despite being trained in buccal data and showing a reduced correlation with chronological age in non-buccal samples, CheekAge was significantly associated with diverse signals in other tissue types [15, 16].

The number of publicly available Infinium MethylationEPIC datasets continues to grow and, as such, we wanted to more comprehensively understand the ability of CheekAge to capture varied disease and health signals in externally collected datasets and compare it to the performance of existing publicly available clocks. Moreover, we wanted to gain insights into the underlying biology driving significant associations. To achieve these aims, we identified and collated 25 different datasets in the Gene Expression Omnibus database [17] that display a significant association with CheekAge or one of five other well-known clocks [10–12, 18, 19]. For every signal significantly associated with CheekAge, we iteratively removed each CpG and re-analyzed the association to identify DNA methylation sites that either promote or hinder the correlation. Finally, we performed enrichment analyses on these CpGs to unveil biological pathways and transcription factor targets connected to these methylation sites and signals.

## Results

### Overview and approach

**Fig. 1:**
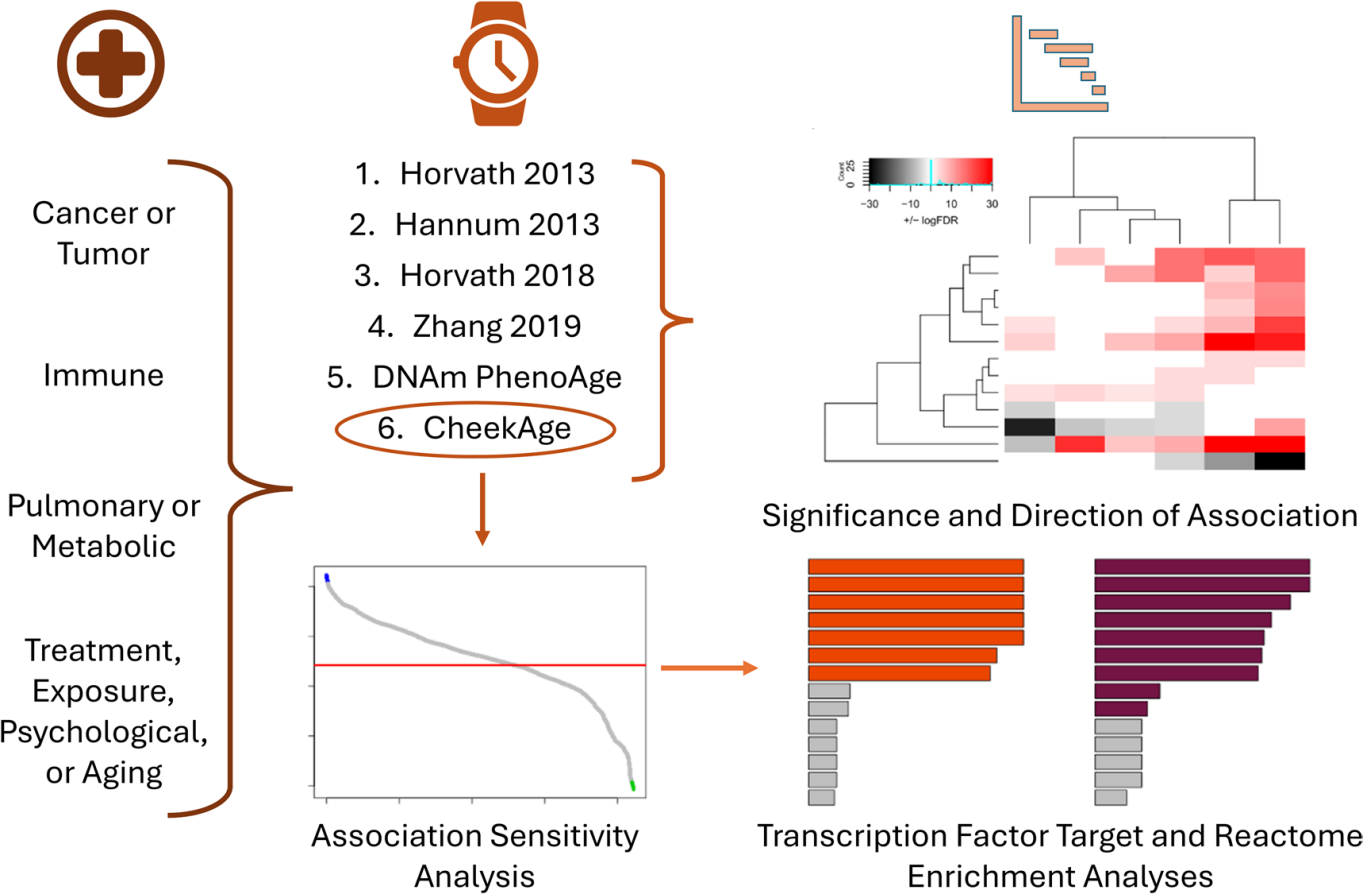
Study overview. In this work, we identified and collated 25 Infinium MethylationEPIC datasets containing chronological age information in the Gene Expression Omnibus database. These datasets—some of which contained multiple signals—were placed into cancer/tumor, immune, pulmonary/metabolic, and other categories. The lattermost “other” category contained aging, psychological, and treatment/exposure signals. The ability of CheekAge and five other clocks (Hannum 2013, Horvath 2013, DNAm PhenoAge, Horvath 2018, and Zhang 2019) to associate with signals across these 25 datasets was then assessed. For each significant association with CheekAge, we iteratively removed each input to examine its impact on the overall association. DNA methylation sites that reduced the significance of the association when removed were dubbed “pro” CpGs while those that increased the significance of the association when omitted were labeled “anti” CpGs. From here, enrichment analyses were performed on “pro” and “anti” CpGs to reveal associated transcription factor targets and Reactome pathways

As visualized in Fig. 1, we looked at the ability of six different epigenetic aging clocks (CheekAge, DNAm PhenoAge, Zhang 2019, Horvath 2018, Horvath 2013, and Hannum 2013) to associate with 46 different signals spanning 25 publicly available Infinium MethylationEPIC datasets by testing the significance of delta age (the difference between epigenetic age and chronological age) against variables of interest. Additional information for each of these clocks can be found in Table 1. Confounding factors such as chronological age, sex/gender, race/ethnicity, and cell-type proportion were included when available. The relevant Gene Expression Omnibus identifiers and variables connected to each dataset can be found in Supplementary Table 1. Broadly speaking, these signals can be placed into four different categories: cancer/tumor, immune, pulmonary/metabolic, and other. The “other” category can be further subdivided into psychological, aging, treatment, and exposure subcategories. For each signal significantly correlated with CheekAge, we iteratively removed each CheekAge CpG input and re-calculated the association to identify DNA methylation sites that either increased (“pro” CpGs) or decreased (“anti” CpGs) the FDR value of an association when removed. From here, detailed enrichment analyses were performed to identify Reactome pathways and transcription factor targets linked to these DNA methylation sites.

**Table 1:** Summary of the clocks used in this study. For each epigenetic aging clock, the name, number of DNA methylation sites used as inputs, tissue type(s) used for training, type of clock (first-generation versus next-generation), and original reference is noted. Clocks are ordered by publication date, in descending order

Epigenetic clock	# of CpG inputs	Tissue(s) used for training	Type of clock	Reference
CheekAge	> 200,000	Buccal	Next-generation	^15^
Zhang 2019	514	Multi-tissue	First-generation	^19^
Horvath 2018	391	Multi-tissue	First-generation	^18^
DNAm PhenoAge	513	Blood	Next-generation	^12^
Horvath 2013	353	Multi-tissue	First-generation	^11^
Hannum 2013	71	Blood	First-generation	^10^

### Cancer and tumor datasets

**Fig. 2:**
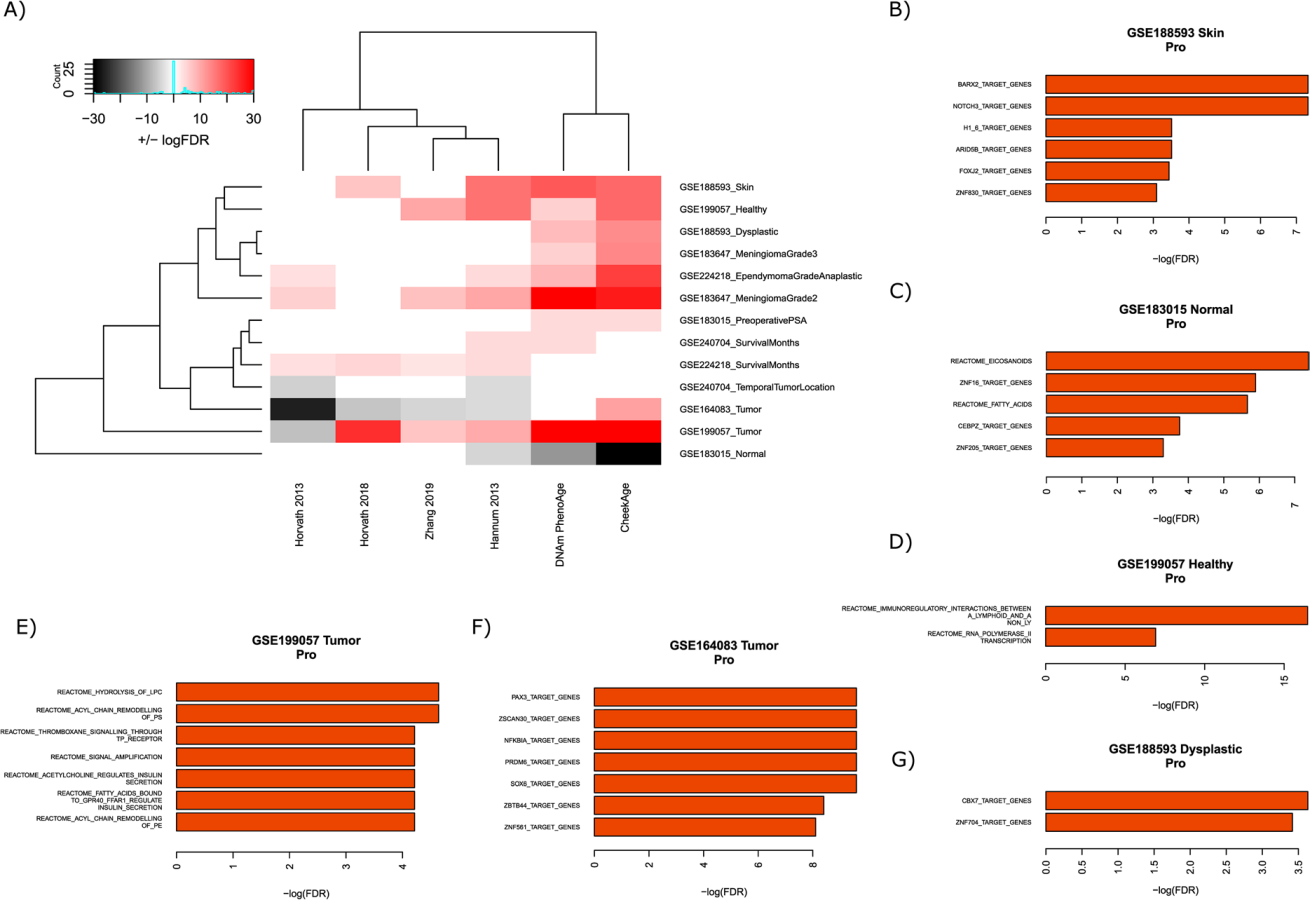
Associations between epigenetic clocks and signals in cancer or tumor datasets. **A** The ability of each clock to associate with distinct signals is visualized. The color white indicates no significant association, the colors pink/red indicate a significant increase in delta age (epigenetic age – chronological age), and the colors gray/black indicate a significant decrease in delta age. Darker colors (red/black) indicate more significant associations than lighter colors (pink/gray). The top transcription factor target and Reactome pathway results for “pro” CpGs are shown for the GSE188593 Skin (**B**), GSE183015 normal (**C**), GSE199057 healthy (**D**), GSE199057 tumor (**E**), GSE164083 tumor (**F**), and GSE188593 dysplastic (**G**) signals

A total of 13 cancer- or tumor-related signals in datasets GSE164083, GSE183015, GSE183647, GSE188593, GSE199057, GSE224218, and GSE240704 were analyzed for their ability to associate with CheekAge and five other epigenetic aging clocks (Fig. 2A). Various cancer and tumor types were represented across these datasets, such as melanocytic nevi, meningioma, ependymoma, glioma, and esophageal squamous cell carcinoma.

The CheekAge, DNAm PhenoAge, and Hannum 2013 clocks each displayed ten significant associations, followed by the Horvath 2013 (six), Zhang 2019 (five), and Horvath 2018 (four) models. While delta age was typically elevated, there were some interesting exceptions. In the esophageal squamous cell carcinoma dataset (GSE164083), delta age was lower for the Horvath 2013, Horvath 2018, Zhang 2019, and Hannum 2013 clocks, and there was no significant correlation with the DNAm PhenoAge clock. The only model that showed a significant increase in delta age was CheekAge (Fig. 2A).

For each of the ten signals significantly associated with CheekAge, each CpG input was iteratively removed to reveal the impact of a given DNA methylation site on the association. For the “pro” CpGs that promote an association, the top transcription factor targets and Reactome pathways for each dataset with significant enrichment results are visually shown (Fig. 2B–G). Significantly enriched Reactome pathways spanned themes like lipid metabolism (“eicosanoids,” “fatty acids,” “hydrolysis of LPC,” “acyl chain remodeling of PS,” and “acyl chain remodeling of PE”), immunity (“immunoregulatory interactions between a lymphoid and a non-lymphoid cell”), transcription (“RNA polymerase II transcription”), and insulin secretion (“acetylcholine regulates insulin secretion” and “fatty acids bound to GPR40 (FFAR1) regulate insulin secretion”). The broader list of enrichment results for “pro” and “anti” CpGs linked to signals related to tumors and cancers can be found in Supplementary Fig. 1.

### Immune datasets

**Fig. 3:**
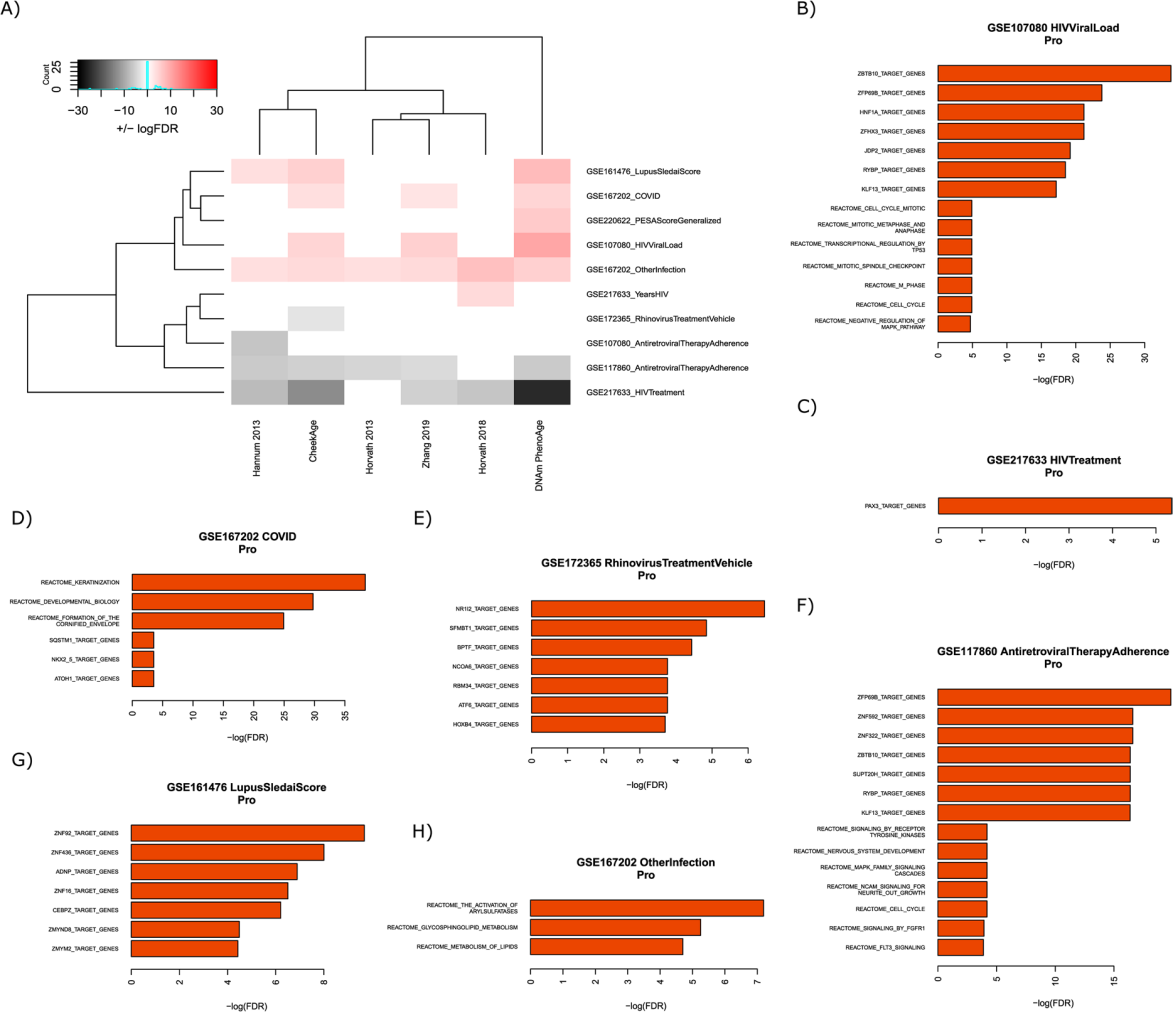
Associations between epigenetic clocks and signals in immune datasets.** A** The ability of each clock to associate with distinct signals is visualized. The color white indicates no significant association, the colors pink/red indicate a significant increase in delta age (epigenetic age – chronological age), and the colors gray/black indicate a significant decrease in delta age. Darker colors (red/black) indicate more significant associations than lighter colors (pink/gray). The top significant transcription factor target and Reactome pathway results for “pro” CpGs are shown for the GSE107080 HIV viral load (**B**), GSE217633 HIV treatment (**C**), GSE167202 COVID (**D**), GSE172365 rhinovirus treatment vehicle (**E**), GSE117860 antiretroviral therapy adherence (**F**), GSE161476 lupus SLEDAI score (**G**), and GSE167202 other infection (**H**) signals

For the “immune” category, we looked at ten distinct signals in the datasets GSE161476, GSE167202, GSE220622, GSE107080, GSE217633, GSE172365, and GSE117860 (Fig. 3A). Examples of these signals include HIV viral load, antiretroviral therapy adherence, the systemic lupus erythematosus disease activity index score, a SARS-CoV-2 infection, and other respiratory infections. CheekAge and DNAm PhenoAge were both associated with seven significant associations, followed by the Hannum 2013 (five), Zhang 2019 (five), Horvath 2018 (three), and Horvath 2013 (two) clocks. One signal corresponding to non-COVID respiratory infections in dataset GSE167202 was significantly associated with epigenetic age acceleration (higher delta age) in all six clocks. In the same dataset, a COVID infection was linked to a higher epigenetic age for the CheekAge, Zhang 2019, and DNAm PhenoAge clocks. In the HIV dataset GSE217633, antiretroviral therapy was associated with epigenetic age deceleration (lower delta age) for all models except the Horvath 2013 clock. In the separate HIV dataset GSE117860, antiretroviral therapy adherence was associated with epigenetic age deceleration in every clock but the Horvath 2018 model.

Numerous Reactome pathways and transcription factor targets were significantly enriched by “pro” CpGs driving CheekAge’s immune associations (Fig. 3B–H). Significant Reactome pathways touched on various motifs, including genomic stability (“cell cycle, mitotic,” “Mitotic metaphase and anaphase,” “transcriptional regulation by TP53,” “mitotic spindle checkpoint,” “M phase,” and “cell cycle”), development (“keratinization,” “developmental biology,” “formation of the cornified envelope,” and “nervous system development”), lipid metabolism (“glycosphingolipid metabolism” and “metabolism of lipids”), and cell signaling (“signaling by receptor tyrosine kinases,” “MAPK family signaling cascades,” “NCAM signaling for neurite out-growth,” “signaling by FGFR1,” and “FLT3 signaling”). More extensive enrichment results for both “pro” and “anti” CpGs connected to immune signals can be found in Supplementary Fig. 2.

### Pulmonary and metabolic datasets

**Fig. 4:**
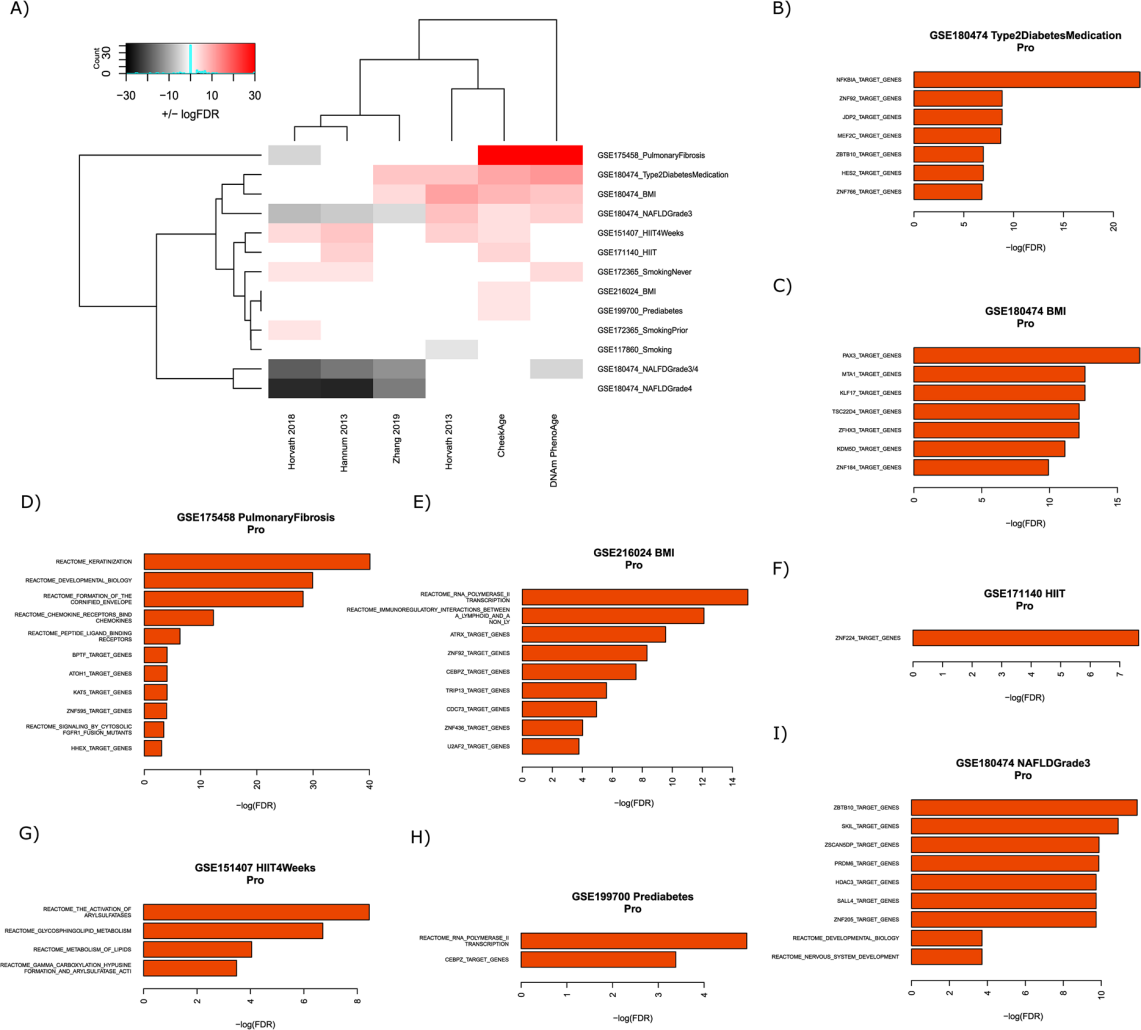
Associations between epigenetic clocks and signals in pulmonary or metabolic datasets.** A** The ability of each clock to associate with distinct signals is visualized. The color white indicates no significant association, the colors pink/red indicate a significant increase in delta age (epigenetic age – chronological age), and the colors gray/black indicate a significant decrease in delta age. Darker colors (red/black) indicate more significant associations than lighter colors (pink/gray). The top transcription factor target and Reactome pathway results for “pro” CpGs are shown for the GSE180474 type 2 diabetes medication (**B)**, GSE180474 BMI (**C**), GSE175458 pulmonary fibrosis (**D**), GSE216024 BMI (**E**), GSE171140 HIIT (**F**), GSE151407 HIIT 4 weeks (**G**), GSE199700 prediabetes (**H**), and GSE180474 NAFLD grade3 (**I**) signals

We next analyzed 13 pulmonary or metabolic signals in the datasets GSE175458, GSE180474, GSE151407, GSE171140, GSE172365, GSE216024, GSE199700, and GSE117860 (Fig. 4A). These signals were eclectic, including variables like prediabetes, body mass index, pulmonary fibrosis, non-alcoholic fatty liver disease, high-intensity interval training, and smoking status (Fig. 4A). CheekAge correlated with a total eight signals, followed by Horvath 2018 (seven), DNAm PhenoAge (six), Hannum 2013 (six), Zhang 2019 (five), and Horvath 2013 (five). The only signal significantly associated with all six clocks was “NAFLDGrade3” in dataset GSE180474. While the CheekAge, DNAm PhenoAge, and Horvath 2013 clocks displayed epigenetic age acceleration for this variable, a lower delta age was seen for Hannum 2013, Horvath 2018, and Zhang 2019 models. The only two clocks that predicted a higher delta age for pulmonary fibrosis were CheekAge and DNAm PhenoAge. High-intensity interval training in the short term was associated with a higher epigenetic age across multiple clocks, which may be reflective of positive inflammatory changes that are known to occur in response to exercise [20].

A plethora of transcription factor target and Reactome pathways were significantly enriched by “pro” CpGs that promote the ability of CheekAge to associate with these pulmonary and metabolic signals (Fig. 4B–I). Collectively, the Reactome pathways were linked to development (“Keratinization,” “Developmental Biology,” “Formation of the cornified envelope,” and “Nervous system development”), immunity (“Chemokine receptors bind chemokines” and “immunoregulatory interactions between a lymphoid and a non-Lymphoid cell”), signaling (“peptide ligand-binding receptors” and “signaling by cytosolic FGFR1 fusion mutants”), transcription (“RNA polymerase II transcription”), lipid metabolism (“glycosphingolipid metabolism” and “metabolism of lipids”), and protein modification (“gamma carboxylation, hypusinylation, hydroxylation, and arylsulfatase activation”). Reactome and transcription target pathways enriched by either “pro” or “anti” pulmonary or metabolic CpGs can be found in Supplementary Fig. 3.

### Aging, psychological, treatment, and exposure datasets

**Fig. 5:**
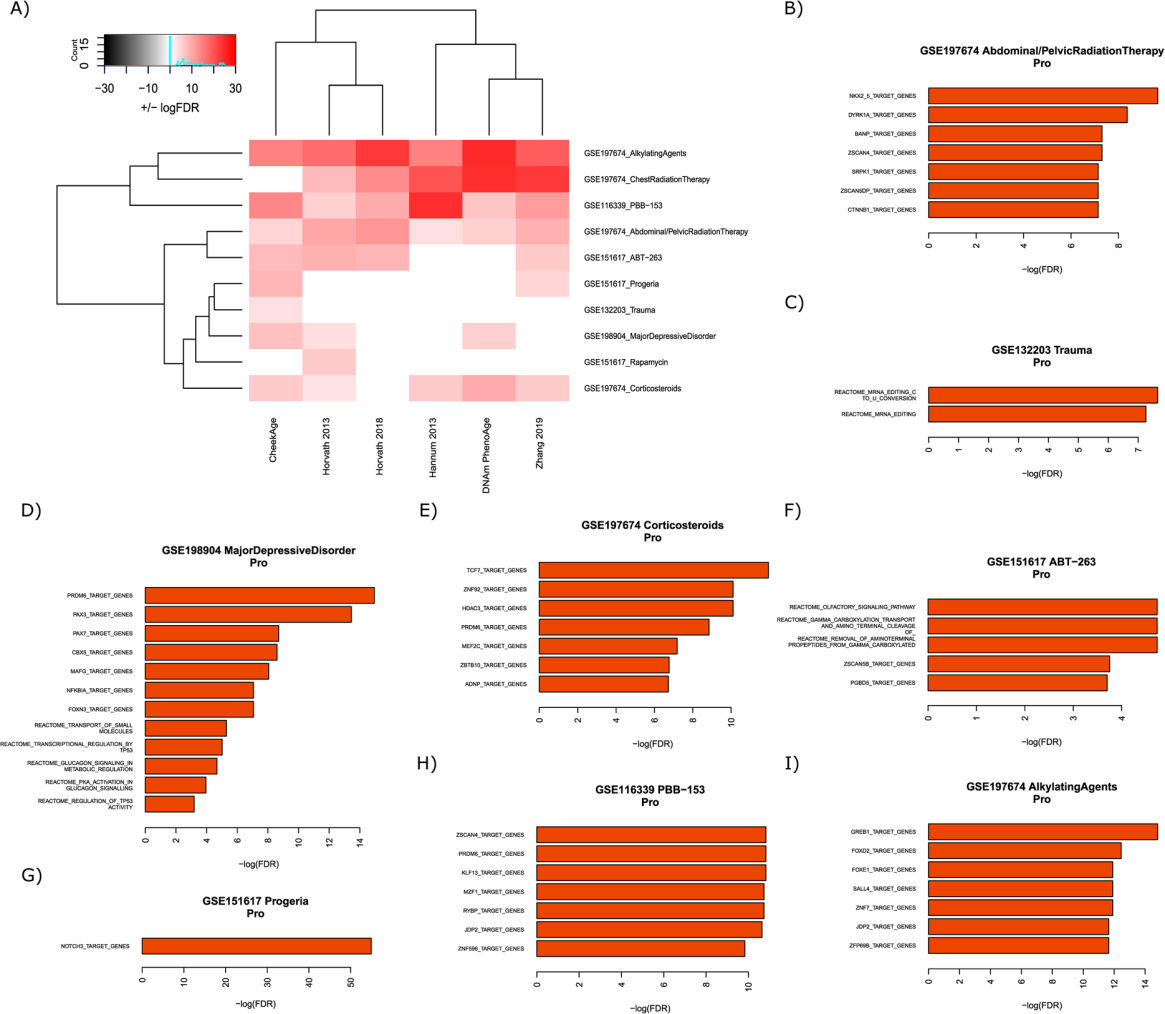
Associations between epigenetic clocks and signals in aging, psychological, treatment, or exposure datasets.** A** The ability of each clock to associate with distinct signals is visualized. The color white indicates no significant association, the colors pink/red indicate a significant increase in delta age (epigenetic age – chronological age), and the colors gray/black indicate a significant decrease in delta age. Darker colors (red/black) indicate more significant associations than lighter colors (pink/gray). The top significant transcription factor target and Reactome pathway results for “pro” CpGs are shown for the GSE197674 abdominal/pelvic radiation therapy (**B**), GSE132203 trauma (C), GSE198904 major depressive disorder (**D**), GSE197674 corticosteroids (**E**), GSE151617 ABT-263 (**F**), GSE151617 progeria (**G**), GSE116339 PBB-153 (**H**), and GSE197674 alkylating agents (**I**) signals

Lastly, we analyzed an eclectic group of 10 signals from datasets GSE197674, GSE116339, GSE197647, GSE151617, GSE132203, and GSE198904 that included psychological trauma, major depressive disorder, the genetic aging disorder progeria, different treatments, and exposure to the industrial chemical PBB-153 (Fig. 5A). The top-performing clocks in this category were CheekAge and the Horvath 2013 clock, each of which displayed a total of eight significant associations. This was followed by the Zhang 2019 (seven), DNAm PhenoAge (six), Hannum 2013 (five), and Horvath 2018 (five) clocks. CheekAge was the only clock to show a significant association (age acceleration) with psychological trauma while the Horvath 2013 clock was only the model to significantly associate (age acceleration) with rapamycin treatment. The latter finding in primary dermal fibroblasts was surprising given that rapamycin extends lifespan in genetically heterogeneous mice [21]. There were three different signals that were significantly associated with a higher delta age for all six clocks, namely exposure to PBB-153, childhood cancer survivors treated with alkylating agents, and childhood cancer survivors treated with abdominal/pelvic radiation therapy. PBB-153, a compound added to plastics to make them less flammable, is a known endocrine disruptor with a long half-life that was inadvertently added to Michigan food systems in the 1970s [22]. Our finding that exposure to this chemical significantly increased epigenetic age according to every clock tested adds to the growing body of literature that certain manufacturing chemicals and byproducts are linked to deleterious effects [23, 24].

A number of Reactome pathways and transcription factor targets were significantly enriched by “pro” CheekAge CpGs responsible for driving identified significant associations (Fig. 5B–I). Taken together, the Reactome results implicated the following biological processes: transcriptional activity (“mRNA editing: C to U conversion” and “mRNA editing”), cell division regulation (“transcriptional regulation by TP53” and “regulation of TP53 activity”), molecular transport (“transport of small molecules”), energy metabolism (“glucagon signaling in metabolic regulation” and “PKA activation in glucagon signaling”), olfaction (“olfactory signaling pathway”), and protein modification (“gamma-carboxylation, transport, and amino-terminal cleavage of proteins” and “removal of aminoterminal propeptides from gamma-carboxylated proteins”). For additional transcription factor target and Reactome pathway results connected to “pro” and “anti” CpGs that impact associations in this category, please see Supplementary Fig. 4.

### Overall clock performance

Visual and tabular summaries of how each clock performed across all signals and datasets can be found in Supplementary Fig. 5 and Table 2, respectively. The clock with the highest number of total significant associations was CheekAge, which correlated with 33 different signals. DNAm PhenoAge displayed 29 significant associations while the Hannum 2013, Zhang 2019, Horvath 2013, and Horvath 2018 epigenetic aging clocks respectively showed 26, 22, 21, and 19 significant associations (Table 2). FDR values and directionality for each association are summarized in Supplementary Table 1. Identified “pro” and “anti” CpGs can be found in Supplementary Information 1 and detailed enrichment results from all analyses are summarized in Supplementary Information 2.

**Table 2:** Total number of clock associations split by category. Clocks are ordered by their total number of associations, from most to least

Epigenetic clock	Cancer/tumor	Immune	Pulmonary/metabolic	Other	Total
CheekAge	10	7	8	8	33
DNAm PhenoAge	10	7	6	6	29
Hannum 2013	10	5	6	5	26
Zhang 2019	5	5	5	7	22
Horvath 2013	6	2	5	8	21
Horvath 2018	4	3	7	5	19

## Discussion

In this study, the clocks with the highest number of significant associations across 25 external datasets were CheekAge and DNAm PhenoAge, two next-generation models. We and others have shown that, when it comes to capturing mortality risk, next-generation clocks are notably more predictive than first-generation clocks [9, 16]. Next-generation clocks have also been shown to be superior when it comes to associating with health signals, such as frailty and walking speed [25]. Furthermore, a recent analysis of publicly available interventional and longitudinal data found that first-generation clocks are less adept at capturing change compared to next-generation clocks [26]. Taken all together, this suggests that researchers should prioritize using more recent, next-generation clocks for health-oriented research.

Very few DNA methylation sites tend to overlap between different epigenetic aging clocks [14, 27] and each model is capturing an aspect of biology [28]. Indeed, in vitro work has found that different epigenetic aging clocks display unique relationships with established hallmarks of aging [29]. In addition to showing that next-generation clocks pick up on more signals overall, we similarly show that each of the six clocks investigated is uniquely capable of associating with signals in a given category. The Hannum 2013 clock, for example, was on par with CheekAge and DNAm PhenoAge in terms of cancer and tumor associations. In comparison, it missed out on multiple associations in the Immune, pulmonary/metabolic, and other categories. DNAm PhenoAge and CheekAge were comparable across the cancer/tumor and immune groups, but CheekAge correlated with two additional signals in each of the remaining categories. In addition, the Horvath 2018 did a decent job at capturing signals in the pulmonary/metabolic category but underperformed in the other categories. These data suggest that disparate clocks should be conceptualized as distinct aging biomarkers that provide different insights into aging biology.

To learn more about the underlying biology of CheekAge, we performed Reactome enrichment analyses on DNA methylation sites that either promoted or antagonized each of CheekAge’s identified associations. We were especially interested in the “pro” CpGs as these were ultimately responsible for CheekAge’s ability to correlate with a given health or disease signal. For the “tumor” variable in the colorectal cancer dataset GSE199057, the Reactome pathways “acetylcholine regulates insulin secretion” and “fatty acids bound to GPR40 (FFAR1) regulate insulin secretion” were both significantly enriched. These results are intriguing given that the insulin/insulin-like growth factor 1 pathway is an evolutionarily conserved regulator of lifespan in model organisms [30] and that lipid metabolism is intimately linked to longevity [31]. Regarding the latter, the pathways “eicosanoids” and “fatty acids” were significantly enriched by the normal signal (associated with a younger epigenetic age according to the Hannum 2013, DNAm PhenoAge, and CheekAge clocks) in dataset GSE183015. For the body mass index signal in dataset GSE216024, the “immunoregulatory interactions between a Lymphoid and a non-lymphoid cell” result was significantly enriched. Not only is a high BMI tethered to immune dysfunction [32], but broad changes to immunity are encompassed by the aging hallmarks chronic inflammation and altered intercellular communication [33]. Some of the more surprising results were observed in the major depressive disorder dataset GSE198904. Specifically, the Reactome pathways “glucagon signaling in metabolic regulation” and “PKA activation in glucagon signaling” were significantly enriched. These results are suggestive of a relationship between depression and metabolic health. Indeed, the incidence of depression is more common in people with diabetes [34] and nascent work suggests that GLP-1 receptor agonists may have antidepressant effects [35].

The Reactome enrichment results from “anti” CpGs—DNA methylation sites that are important for the CheekAge clock but actually improve a given association when removed—also provide novel biological insights. In the esophageal squamous cell carcinoma dataset (GSE164083), the pathway “DNA damage/telomere stress induced senescence” was significantly enriched by the relevant “anti” CpGs. Not only is cellular senescence one of the 12 hallmarks of aging, but DNA damage and telomere stress are highly germane to the aging hallmarks genomic instability and telomere attrition [33]. The distinct pathways “adaptive immune system” and “antimicrobial peptides” were significantly enriched in the HIV dataset GSE107080 and the respiratory infection dataset GSE167202, respectively. In our previous work involving proteomic aging clocks, we observed that clock inputs were frequently linked to immune system pathways [36]. We also showed that proteomic clocks composed of proteins that are present in the Reactome “adaptive immune system” pathway or other immune pathways were especially good at predicting age [36]. The result “SUMOylation of DNA methylation proteins” in the prediabetes dataset (GSE199700) is also worth noting given its implication of epigenetic regulation. In the major depressive disorder dataset GSE198904, the pathway “laminin interactions” was significantly enriched by “anti” CpGs. This result is intriguing given the growing recognition that the extracellular matrix deteriorates with age and that this degradation likely contributes to the aging process [37]. Moreover, an increased risk of early mortality has been observed in patients with major depressive disorder [38].

In addition to looking at Reactome, we also analyzed which transcription factor target genes were significantly enriched by “pro” and “anti” CpGs. For the “dysplastic” signal in skin cancer dataset GSE188593, the two significant transcription factor results enriched by “pro” CpGs were “CBX7 target genes” and “ZNF704 target genes.” *CBX7* has been shown to play multiple roles in cancer by inhibiting oncosuppressor genes and modulating the expression of cell-cycle proteins [39]. Less is known about *ZNF704*, though early research has linked it to uveal melanoma [40], chondrosarcoma [41], and breast cancer [42]. Moreover, a genome-wide association study identified *ZNF704* as a gene candidate associated with healthy aging [43]. Turning to the rhinovirus dataset GSE172365, one of the significant transcription factor results enriched by “pro” CpGs was “ATF6 target genes.” In addition to mediating the unfolded protein response, the *Caenorhabditis elegans* ortholog of this gene (*atf-6*) was shown to regulate worm lifespan by modulating calcium homeostasis in mitochondria [44]. One transcription factor that showed up several times across “pro” and “anti” CpG enrichment analyses was *KLF13*. Mutations in this gene underlie a form of congenital heart disease [45] and cg07814318, a CpG site annotated to *KLF13*, is associated with both obesity and obesity-related traits [46]. A recent study also showed that this gene stimulates proinflammatory cytokines and facilitates immune activation [47]. “NFKBIA target genes” was another recurrent result that implicates the gene *NFKBIA*, which encodes for NF-kappa-B inhibitor alpha. A prior report showed that mutating this gene in mouse endothelium protected against insulin resistance and prolonged life [48]. For the progeria signal in dataset GSE151617, the sole result was “NOTCH3 target genes.” Not only does signaling of this transcription factor starkly decline with age in mice and human brain vessels, but mice lacking *Notch3* develop glymphatic dysfunction and neurodegeneration [49]. Many of the implicated transcription factors were also listed in the human ageing genomic resources (HAGR) collection of databases [50]. For example, one of the results enriched by “pro” CpGs in the GSE167202 COVID dataset was “SQSTM1 target genes.” One of the reasons *SQSTM1* is listed in HAGR is because mice lacking this gene display an accelerated aging phenotype characterized by mitochondrial dysfunction and a shorter lifespan [51].

As research tools, next-generation (or health-trained) epigenetic aging clocks like CheekAge display an impressive ability to associate with diverse health variables. Given that they are providing unique insights into the aging epigenome and that epigenetic alterations represent a fundamental aging hallmark [33], there is utility in incorporating these clocks and future versions of these models into more clinical trials. Not only would this give us more insights into the kinds of interventions that slow next-generation clocks, but these trials would teach us about which models and methylomic inputs are more or less suited for interventional testing. It would also be worth determining if current or subsequent models can enhance the predictive power of existing risk calculators routinely used by clinicians. Furthermore, approaches that increase the test–retest reliability of epigenetic clocks will further increase their potential utility. While we and others have reported that clustering [15], ensembling [15], and principal component analysis [52] can significantly increase reproducibility, future research is warranted to see if further improvements are possible. It remains to be determined, for example, if technical reproducibility is comparable between array-based and sequencing-based techniques for producing methylomic data.

In summary, this work provides further evidence that next-generation clocks are superior to first-generation clocks when it comes to capturing health and disease signals. In addition, we demonstrate that CheekAge is capable of associating with a wide range of variables affecting a range of organ systems. We also gain novel insights into the biology connected to the CpG sites that make up CheekAge, identifying myriad connections to age-related processes and transcription factors. Additional research is warranted to better understand CheekAge’s ability to capture change in longitudinal and interventional datasets. Via DNA methylation editing [53], it would also be worthwhile to determine the experimental impact of manipulating key DNA methylation sites that drive associations with clinical variables. Finally, it would be valuable to understand how other epigenetic aging clocks, such as DNAmFitAge [54] and different pan-mammalian clocks [55], perform in the 25 Infinium MethylationEPIC datasets we have collated.

## Methods

### Dataset selection and preprocessing

We collated 25 methylation array datasets from the Gene Expression Omnibus [17] for our association studies and analyses. We selected Infinium MethylationEPIC array datasets (platform GPL21145) since it included coverage of all CpGs needed for all six epigenetic clocks used. Furthermore, we selected datasets which included chronological age information, which was necessary for delta age calculations. Finally, we selected datasets with at least 50 samples and with health, disease, or lifestyle variables measured. While these filters did limit the total of number of datasets and variables we could analyze, they helped ensure that all clocks could be utilized and that enough samples were present to detect significant associations with delta age. Datasets were preprocessed using the minfi package version 1.46 [56]. Raw IDATs were read in and normalized using the preprocessNoob function, and beta methylation values were calculated with the getBeta function. When IDAT files were not available, signal intensity files were used and converted into methylation set objects compatible with the minfi workflow (GSE117860 and GSE198904). When IDATs or methylation signals were not available, processed methylation values were used directly (GSE107080). The full list of methylation datasets used is available in the “Data Availability” section and in Supplementary Table 1.

### Epigenetic age prediction

We selected five well-known, publicly available clocks from the methyl CIPHER package [57]. There were four first-generation clocks included: Hannum 2013 [10], Horvath 2013 [11], Horvath 2018 [18], and Zhang 2019 [19]. In addition, the next-generation model DNAm PhenoAge was included [12]. Age prediction for these five clocks was run on the preprocessed beta methylation values from each dataset using the calcUserClocks methyl CIPHER function. CheekAge was predicted following the same workflow as described previously [15]. Briefly beta methylation values were converted to M values:

$${M}_{i}={\text{log}}_{2}\left(\frac{{beta}_{i}}{1-{beta}_{i}}\right),$$

where *M*_*i*_ is the *i*th *M* value, and beta_*i*_ is the *i*th beta value bound to be between 0.00001 and 0.99999 to avoid infinities. Then, *M* values were averaged across CheekAge methylation clusters. An ensemble of linear models was then used to predict an average age, weighted by the training optimization minimum of each linear model in the ensemble, resulting in a CheekAge estimate for each sample. Importantly, since there were differences in the dataset tissue type and preprocessing, and since each clock was trained on a different set of samples and tissues (Table 1), the exact age prediction was converted to a delta age, the difference between the predicted age and the chronological age of each sample. This delta age was used for linear model fitting and testing for significant associations. Batch effect correction was not performed since these datasets were analyzed independently from each other and since only the difference in a variable of interest (e.g., disease) within each dataset was analyzed.

### Identifying significant associations

In order to identify significant associations in each dataset, we constructed an additive linear model for delta age using all available variables, including confounding variables such as chronological age, sex/gender, race/ethnicity, and cell-type proportions. When multiple variables of a similar type were available that were highly correlated or anti-correlated, typically only one was kept. For example, when including cell type proportions, typically just one cell type was included as a confounding variable in the model. For blood-based datasets, when cell type proportion was not available, cell type was estimated using the EpiDISH R package v 2.16.0 [58], which was applicable to GSE116339, GSE217633, and GSE220622. A linear model was constructed using the R lm function, and the Benjamini-Hotchberg multiple-testing correction [59] was applied to the T statistics for significance using the p.adjust R function with the “FDR” method. A FDR threshold value of 0.05 was used to identify significantly associated variables for each clock and CheekAge was further subjected to association sensitivity and functional enrichment analyses. All metadata can be found in Supplementary Table 2. All linear model designs and resulting statistics can be found in Supplementary Table 3.

### Association sensitivity and functional enrichment analyses

Since CheekAge uses over 200,000 CpG inputs to predict age, we wanted to learn more about which CpGs and potential biological processes are important for associations across datasets. To do this, we systematically removed each CheekAge CpG and recalculated CheekAge, delta CheekAge, and the FDR of the association for each variable in each dataset. We then ranked the CpGs based on the FDR value of the association when it was removed, with the top 1000 CpGs that increased FDR for an association classified as “pro” association CpGs, and the bottom 1000 CPGs that decreased FDR for an association classified as “anti” association CpGs. The resulting top 1000 and bottom 1000 CpGs were then subjected to functional enrichment analyses using the missMethyl R package v 1.34.0 [60]. We tested overrepresentation of genes annotated to the top 1000 and bottom 1000 CpGs using both the transcription factor target gene database (1115 gene sets) and the Reactome database v89 (1736 gene sets) from the molecular signatures database [61]. The exact gene sets used for enrichment analysis are included as Supplementary Information 3 and Supplementary Information 4. For the top 1000 and bottom 1000 CheekAge CpGs, please see Supplementary Information 1. For the top 20 enrichment results along with enrichment statistics, please see Supplementary Information 2.

### Analysis visualization

Heatmaps were generated using the heatmap.2 function from the R gplots package v 3.1.3 (https://cran.r-project.org/web/packages/gplots/index.html). The negative log of the FDR was multiplied by the sign of the variable estimate for each linear model and values less than 3 (representing FDR values > 0.05) set to 0 (white), and values capped at ± 30 to de-emphasize outliers. For exact FDR values and association statistics, please see Supplementary Table 3. The negative log of the FDR for Reactome and transcription factor target gene sets for both “pro” and “anti” association CpGs was plotted using the barplot function in R. The main figures show a combination of the top 7 significant Reactome and the top 7 significant transcription factor target enrichment results (FDR < 0.05) for only the “pro” association CheekAge CpGs. The supplemental figures show a combination of the top 7 significant Reactome and the top 7 significant transcription factor target enrichment results for both the “pro” and “anti” association CheekAge CpGs, with colored bars indicating significant associations. Bar plots are shown when at least one significant enrichment result was found.

## Supplementary Information

Below is the link to the electronic supplementary material.

Supplementary Fig. 1 Enrichment plots for cancer and tumor datasets. Transcription factor target and Reactome pathway enrichment results are shown for both “pro” and “anti” CpG groups that respectively promote or antagonize CheekAge’s ability to associate with signals in cancer and tumor datasets. Significant results for “pro” CpGs are shown in orange while significant results for “anti” CpGs are shown in purple. Non-significant results are colored grey (PDF 12 KB)

Supplementary Fig. 2 Enrichment plots for immune datasets. Transcription factor target and Reactome pathway enrichment results are shown for both “pro” and “anti” CpG groups that respectively promote or antagonize CheekAge’s ability to associate with signals in immune datasets. Significant results for “pro” CpGs are shown in orange while significant results for “anti” CpGs are shown in purple. Non-significant results are colored grey (PDF 11 KB)

Supplementary Fig. 3 Enrichment plots for pulmonary and metabolic datasets. Transcription factor target and Reactome pathway enrichment results are shown for both “pro” and “anti” CpG groups that respectively promote or antagonize CheekAge’s ability to associate with signals in pulmonary and metabolic datasets. Significant results for “pro” CpGs are shown in orange while significant results for “anti” CpGs are shown in purple. Non-significant results are colored grey (PDF 12 KB)

Supplementary Fig. 4 Enrichment plots for aging, psychological, drug, treatment, and exposure datasets. Transcription factor target and Reactome pathway enrichment results are shown for both “pro” and “anti” CpG groups that respectively promote or antagonize CheekAge’s ability to associate with signals in aging, psychological, drug, treatment, and exposure datasets. Significant results for “pro” CpGs are shown in orange while significant results for “anti” CpGs are shown in purple. Non-significant results are colored grey (PDF 12 KB)

Supplementary Fig. 5 Summary of association results for all clocks and datasets. The ability of each clock to associate with distinct signals is visualized. The color white indicates no significant association, the colors pink/red indicate a significant increase in delta age (epigenetic age – chronological age), and the colors grey/black indicate a significant decrease in delta age. Darker colors (red/black) indicate more significant associations than lighter colors (pink/grey) (PDF 36 KB)

Supplementary Table 1 (XLSX 49 KB)

Supplementary Table 2 (XLSX 3120 KB)

Supplementary Table 3 (XLSX 99 KB)

Supplementary Information 1 (TXT 994 KB)

Supplementary Information 2 (TXT 1471 KB)

Supplementary Information 3 (TXT 1683 KB)

Supplementary Information 4 (TXT 528 KB)

## Data Availability

All of the publicly available datasets used in this study can be found in the Gene Expression Omnibus database using the following identifiers: GSE107080, GSE116339, GSE117860, GSE132203, GSE151407, GSE151617, GSE161476, GSE164083, GSE167202, GSE171140, GSE172365, GSE175458, GSE180474, GSE183015, GSE183647, GSE188593, GSE197674, GSE198904, GSE199057, GSE199700, GSE216024, GSE217633, GSE220622, GSE224218, and GSE240704. As an alternative to directly downloading and processing these datasets from the Gene Expression Omnibus database, we are happy to provide the metadata and beta values for these 25 datasets upon request. Our previously created ShinyApp, which can be accessed via the subsequent link, can be used to run CheekAge on compatible methylomic data as well as explore CpG-level associations in a large buccal dataset: https://cheekage.tallyhealth.com/.
