# Supplementary material for: Various diseases and conditions are strongly associated with the next-generation epigenetic aging clock CheekAge
Source: GeroScience. 2025 Mar 7;47(3):3191–206. doi: 10.1007/s11357-025-01579-9 (PMC12181163; doi:10.1007/s11357-025-01579-9)
Supplement: Supplementary file 1 — Supplementary Fig. 1 Enrichment plots for cancer and tumor datasets. Transcription factor target and Reactome pathway enrichment results are shown for both “pro” and “anti” CpG groups that respectively promote or antagonize CheekAge’s ability to associate with signals in cancer and tumor datasets. Significant results for “pro” CpGs are shown in orange while significant results for “anti” CpGs are shown in purple. Non-significant results are colored grey (PDF 12 KB) [file 11357_2025_1579_MOESM1_ESM.pdf]

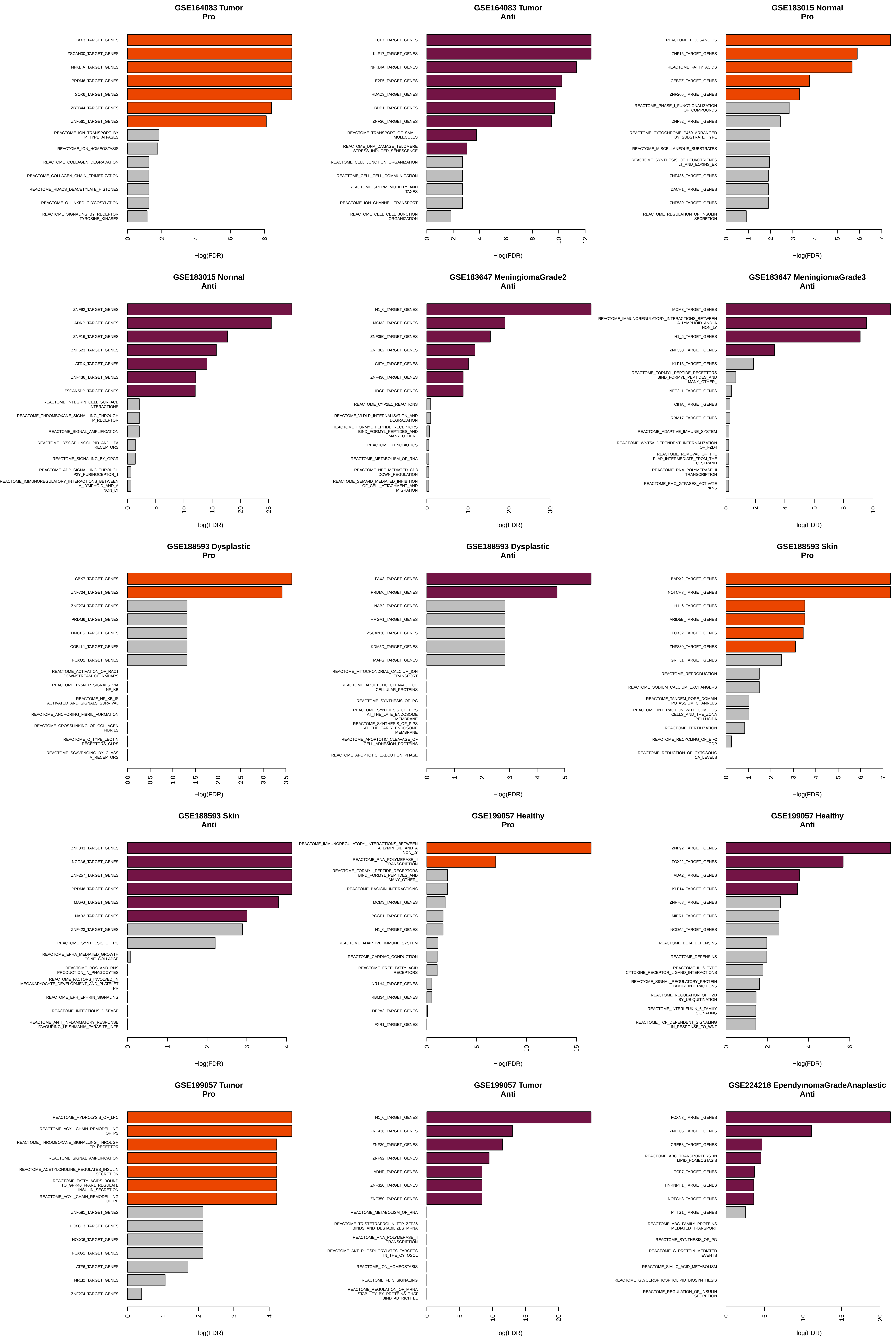

GSE164083 Tumor
Pro
PAX3_TARGET_GENES
ZSCAN30_TARGET_GENES
NFKBIA_TARGET_GENES
PRDM6_TARGET_GENES
SOX6_TARGET_GENES
ZBTB44_TARGET_GENES
ZNF561_TARGET_GENES
REACTOME_ION_TRANSPORT_BY P_TYPE_ATPASES
REACTOME_ION_HOMEOSTASIS
REACTOME_COLLAGEN_DEGRADATION
REACTOME_COLLAGEN_CHAIN_TRIMERIZATION
REACTOME_HDACS_DEACETYLATE_HISTONES
REACTOME_O_LINKED_GLYCOSYLATION
REACTOME_SIGNALING_BY_RECEPTOR TYROSINE_KINASES
0 2 4 6 8
−log(FDR)
GSE164083 Tumor
Anti
TCF7_TARGET_GENES
KLF17_TARGET_GENES
NFKBIA_TARGET_GENES
E2F5_TARGET_GENES
HDAC3_TARGET_GENES
BDP1_TARGET_GENES
ZNF30_TARGET_GENES
REACTOME_TRANSPORT_OF_SMALL MOLECULES
REACTOME_DNA_DAMAGE_TELOMERE STRESS_INDUCED_SENESCENCE
REACTOME_CELL_JUNCTION_ORGANIZATION
REACTOME_CELL_CELL_COMMUNICATION
REACTOME_SPERM_MOTILITY_AND TAXES
REACTOME_ION_CHANNEL_TRANSPORT
REACTOME_CELL_CELL_JUNCTION ORGANIZATION
0 2 4 6 8 10 12
−log(FDR)
GSE183015 Normal
Pro
REACTOME_EICOSANOIDS
ZNF16_TARGET_GENES
REACTOME_FATTY_ACIDS
CEBPZ_TARGET_GENES
ZNF205_TARGET_GENES
REACTOME_PHASE_I_FUNCTIONALIZATION OF_COMPOUNDS
ZNF92_TARGET_GENES
REACTOME_CYTOCHROME_P450_ARRANGED BY_SUBSTRATE_TYPE
REACTOME_MISCELLANEOUS_SUBSTRATES
REACTOME_SYNTHESIS_OF_LEUKOTRIENES LT_AND_EOXINS_EX
ZNF436_TARGET_GENES
DACH1_TARGET_GENES
ZNF589_TARGET_GENES
REACTOME_REGULATION_OF_INSULIN SECRETION
0 1 2 3 4 5 6 7
−log(FDR)
GSE183015 Normal
Anti
ZNF92_TARGET_GENES
ADNP_TARGET_GENES
ZNF16_TARGET_GENES
ZNF623_TARGET_GENES
ATRX_TARGET_GENES
ZNF436_TARGET_GENES
ZSCAN5DP_TARGET_GENES
REACTOME_INTEGRIN_CELL_SURFACE INTERACTIONS
REACTOME_THROMBOXANE_SIGNALLING_THROUGH TP_RECEPTOR
REACTOME_SIGNAL_AMPLIFICATION
REACTOME_LYSOSPHINGOLIPID_AND_LPA RECEPTORS
REACTOME_SIGNALING_BY_GPCR
REACTOME_ADP_SIGNALLING_THROUGH P2Y_PURINOCEPTOR_1
REACTOME_IMMUNOREGULATORY_INTERACTIONS_BETWEEN A_LYMPHOID_AND_A NON_LY
0 5 10 15 20 25
−log(FDR)
GSE183647 MeningiomaGrade2
Anti
H1_6_TARGET_GENES
MCM3_TARGET_GENES
ZNF350_TARGET_GENES
ZNF362_TARGET_GENES
CIITA_TARGET_GENES
ZNF436_TARGET_GENES
HDGF_TARGET_GENES
REACTOME_CYP2E1_REACTIONS
REACTOME_VLDLR_INTERNALISATION_AND DEGRADATION
REACTOME_FORMYL_PEPTIDE_RECEPTORS BIND_FORMYL_PEPTIDES_AND MANY_OTHER_
REACTOME_XENOBIOTICS
REACTOME_METABOLISM_OF_RNA
REACTOME_NEF_MEDIATED_CD8 DOWN_REGULATION
REACTOME_SEMA4D_MEDIATED_INHIBITION OF_CELL_ATTACHMENT_AND MIGRATION
0 10 20 30
−log(FDR)
GSE183647 MeningiomaGrade3
Anti
MCM3_TARGET_GENES
REACTOME_IMMUNOREGULATORY_INTERACTIONS_BETWEEN A_LYMPHOID_AND_A NON_LY
H1_6_TARGET_GENES
ZNF350_TARGET_GENES
KLF13_TARGET_GENES
REACTOME_FORMYL_PEPTIDE_RECEPTORS BIND_FORMYL_PEPTIDES_AND MANY_OTHER_
NFE2L1_TARGET_GENES
CIITA_TARGET_GENES
RBM17_TARGET_GENES
REACTOME_ADAPTIVE_IMMUNE_SYSTEM
REACTOME_WNT5A_DEPENDENT_INTERNALIZATION OF_FZD4
REACTOME_REMOVAL_OF_THE FLAP_INTERMEDIATE_FROM_THE C_STRAND
REACTOME_RNA_POLYMERASE_II TRANSCRIPTION
REACTOME_RHO_GTPASES_ACTIVATE PKNS
0 2 4 6 8 10
−log(FDR)
GSE188593 Dysplastic
Pro
CBX7_TARGET_GENES
ZNF704_TARGET_GENES
ZNF274_TARGET_GENES
PRDM6_TARGET_GENES
HMCES_TARGET_GENES
COBLL1_TARGET_GENES
FOXQ1_TARGET_GENES
REACTOME_ACTIVATION_OF_RAC1 DOWNSTREAM_OF_NMDARS
REACTOME_P75NTR_SIGNALS_VIA NF_KB
REACTOME_NF_KB_IS ACTIVATED_AND_SIGNALS_SURVIVAL
REACTOME_ANCHORING_FIBRIL_FORMATION
REACTOME_CROSSLINKING_OF_COLLAGEN FIBRILS
REACTOME_C_TYPE_LECTIN RECEPTORS_CLRS
REACTOME_SCAVENGING_BY_CLASS A_RECEPTORS
0.0 0.5 1.0 1.5 2.0 2.5 3.0 3.5
−log(FDR)
GSE188593 Dysplastic
Anti
PAX3_TARGET_GENES
PRDM6_TARGET_GENES
NAB2_TARGET_GENES
HMGA1_TARGET_GENES
ZSCAN30_TARGET_GENES
KDM5D_TARGET_GENES
MAFG_TARGET_GENES
REACTOME_MITOCHONDRIAL_CALCIUM_ION TRANSPORT
REACTOME_APOPTOTIC_CLEAVAGE_OF CELLULAR_PROTEINS
REACTOME_SYNTHESIS_OF_PC
REACTOME_SYNTHESIS_OF_PIPS AT_THE_LATE_ENDOSOME MEMBRANE
REACTOME_SYNTHESIS_OF_PIPS AT_THE_EARLY_ENDOSOME MEMBRANE
REACTOME_APOPTOTIC_CLEAVAGE_OF CELL_ADHESION_PROTEINS
REACTOME_APOPTOTIC_EXECUTION_PHASE
0 1 2 3 4 5
−log(FDR)
GSE188593 Skin
Pro
BARX2_TARGET_GENES
NOTCH3_TARGET_GENES
H1_6_TARGET_GENES
ARID5B_TARGET_GENES
FOXJ2_TARGET_GENES
ZNF830_TARGET_GENES
GRHL1_TARGET_GENES
REACTOME_REPRODUCTION
REACTOME_SODIUM_CALCIUM_EXCHANGERS
REACTOME_TANDEM_PORE_DOMAIN POTASSIUM_CHANNELS
REACTOME_INTERACTION_WITH_CUMULUS CELLS_AND_THE_ZONA PELLUCIDA
REACTOME_FERTILIZATION
REACTOME_RECYCLING_OF_EIF2 GDP
REACTOME_REDUCTION_OF_CYTOSOLIC CA_LEVELS
0 1 2 3 4 5 6 7
−log(FDR)
GSE188593 Skin
Anti
ZNF843_TARGET_GENES
NCOA6_TARGET_GENES
ZNF257_TARGET_GENES
PRDM6_TARGET_GENES
MAFG_TARGET_GENES
NAB2_TARGET_GENES
ZNF423_TARGET_GENES
REACTOME_SYNTHESIS_OF_PC
REACTOME_EPHA_MEDIATED_GROWTH CONE_COLLAPSE
REACTOME_ROS_AND_RNS PRODUCTION_IN_PHAGOCYTES
REACTOME_FACTORS_INVOLVED_IN MEGAKARYOCYTE_DEVELOPMENT_AND_PLATELET PR
REACTOME_EPH_EPHRIN_SIGNALING
REACTOME_INFECTIOUS_DISEASE
REACTOME_ANTI_INFLAMMATORY_RESPONSE FAVOURING_LEISHMANIA_PARASITE_INFE
0 1 2 3 4
−log(FDR)
GSE199057 Healthy
Pro
REACTOME_IMMUNOREGULATORY_INTERACTIONS_BETWEEN A_LYMPHOID_AND_A NON_LY
REACTOME_RNA_POLYMERASE_II TRANSCRIPTION
REACTOME_FORMYL_PEPTIDE_RECEPTORS BIND_FORMYL_PEPTIDES_AND MANY_OTHER_
REACTOME_BASIGIN_INTERACTIONS
MCM3_TARGET_GENES
PCGF1_TARGET_GENES
H1_6_TARGET_GENES
REACTOME_ADAPTIVE_IMMUNE_SYSTEM
REACTOME_CARDIAC_CONDUCTION
REACTOME_FREE_FATTY_ACID RECEPTORS
NR1H4_TARGET_GENES
RBM34_TARGET_GENES
DPPA3_TARGET_GENES
FXR1_TARGET_GENES
0 5 10 15
−log(FDR)
GSE199057 Healthy
Anti
ZNF92_TARGET_GENES
FOXJ2_TARGET_GENES
ADA2_TARGET_GENES
KLF14_TARGET_GENES
ZNF768_TARGET_GENES
MIER1_TARGET_GENES
NCOA4_TARGET_GENES
REACTOME_BETA_DEFENSINS
REACTOME_DEFENSINS
REACTOME_IL_6_TYPE CYTOKINE_RECEPTOR_LIGAND_INTERACTIONS
REACTOME_SIGNAL_REGULATORY_PROTEIN FAMILY_INTERACTIONS
REACTOME_REGULATION_OF_FZD BY_UBIQUITINATION
REACTOME_INTERLEUKIN_6_FAMILY SIGNALING
REACTOME_TCF_DEPENDENT_SIGNALING IN_RESPONSE_TO_WNT
0 2 4 6
−log(FDR)
GSE199057 Tumor
Pro
REACTOME_HYDROLYSIS_OF_LPC
REACTOME_ACYL_CHAIN_REMODELLING OF_PS
REACTOME_THROMBOXANE_SIGNALLING_THROUGH TP_RECEPTOR
REACTOME_SIGNAL_AMPLIFICATION
REACTOME_ACETYLCHOLINE_REGULATES_INSULIN SECRETION
REACTOME_FATTY_ACIDS_BOUND TO_GPR40_FFAR1_REGULATE INSULIN_SECRETION
REACTOME_ACYL_CHAIN_REMODELLING OF_PE
ZNF581_TARGET_GENES
HOXC13_TARGET_GENES
HOXC6_TARGET_GENES
FOXG1_TARGET_GENES
ATF6_TARGET_GENES
NR1I2_TARGET_GENES
ZNF274_TARGET_GENES
0 1 2 3 4
−log(FDR)
GSE199057 Tumor
Anti
H1_6_TARGET_GENES
ZNF436_TARGET_GENES
ZNF30_TARGET_GENES
ZNF92_TARGET_GENES
ADNP_TARGET_GENES
ZNF320_TARGET_GENES
ZNF350_TARGET_GENES
REACTOME_METABOLISM_OF_RNA
REACTOME_TRISTETRAPROLIN_TTP_ZFP36 BINDS_AND_DESTABILIZES_MRNA
REACTOME_RNA_POLYMERASE_II TRANSCRIPTION
REACTOME_AKT_PHOSPHORYLATES_TARGETS IN_THE_CYTOSOL
REACTOME_ION_HOMEOSTASIS
REACTOME_FLT3_SIGNALING
REACTOME_REGULATION_OF_MRNA STABILITY_BY_PROTEINS_THAT BIND_AU_RICH_EL
0 5 10 15 20
−log(FDR)
GSE224218 EpendymomaGradeAnaplastic
Anti
FOXN3_TARGET_GENES
ZNF205_TARGET_GENES
CREB3_TARGET_GENES
REACTOME_ABC_TRANSPORTERS_IN LIPID_HOMEOSTASIS
TCF7_TARGET_GENES
HNRNPH1_TARGET_GENES
NOTCH3_TARGET_GENES
PTTG1_TARGET_GENES
REACTOME_ABC_FAMILY_PROTEINS MEDIATED_TRANSPORT
REACTOME_SYNTHESIS_OF_PG
REACTOME_G_PROTEIN_MEDIATED EVENTS
REACTOME_SIALIC_ACID_METABOLISM
REACTOME_GLYCEROPHOSPHOLIPID_BIOSYNTHESIS
REACTOME_REGULATION_OF_INSULIN SECRETION
0 5 10 15 20
−log(FDR)
