# Supplementary material for: Various diseases and conditions are strongly associated with the next-generation epigenetic aging clock CheekAge
Source: GeroScience. 2025 Mar 7;47(3):3191–206. doi: 10.1007/s11357-025-01579-9 (PMC12181163; doi:10.1007/s11357-025-01579-9)
Supplement: Supplementary file 2 — Supplementary Fig. 2 Enrichment plots for immune datasets. Transcription factor target and Reactome pathway enrichment results are shown for both “pro” and “anti” CpG groups that respectively promote or antagonize CheekAge’s ability to associate with signals in immune datasets. Significant results for “pro” CpGs are shown in orange while significant results for “anti” CpGs are shown in purple. Non-significant results are colored grey (PDF 11 KB) [file 11357_2025_1579_MOESM2_ESM.pdf]

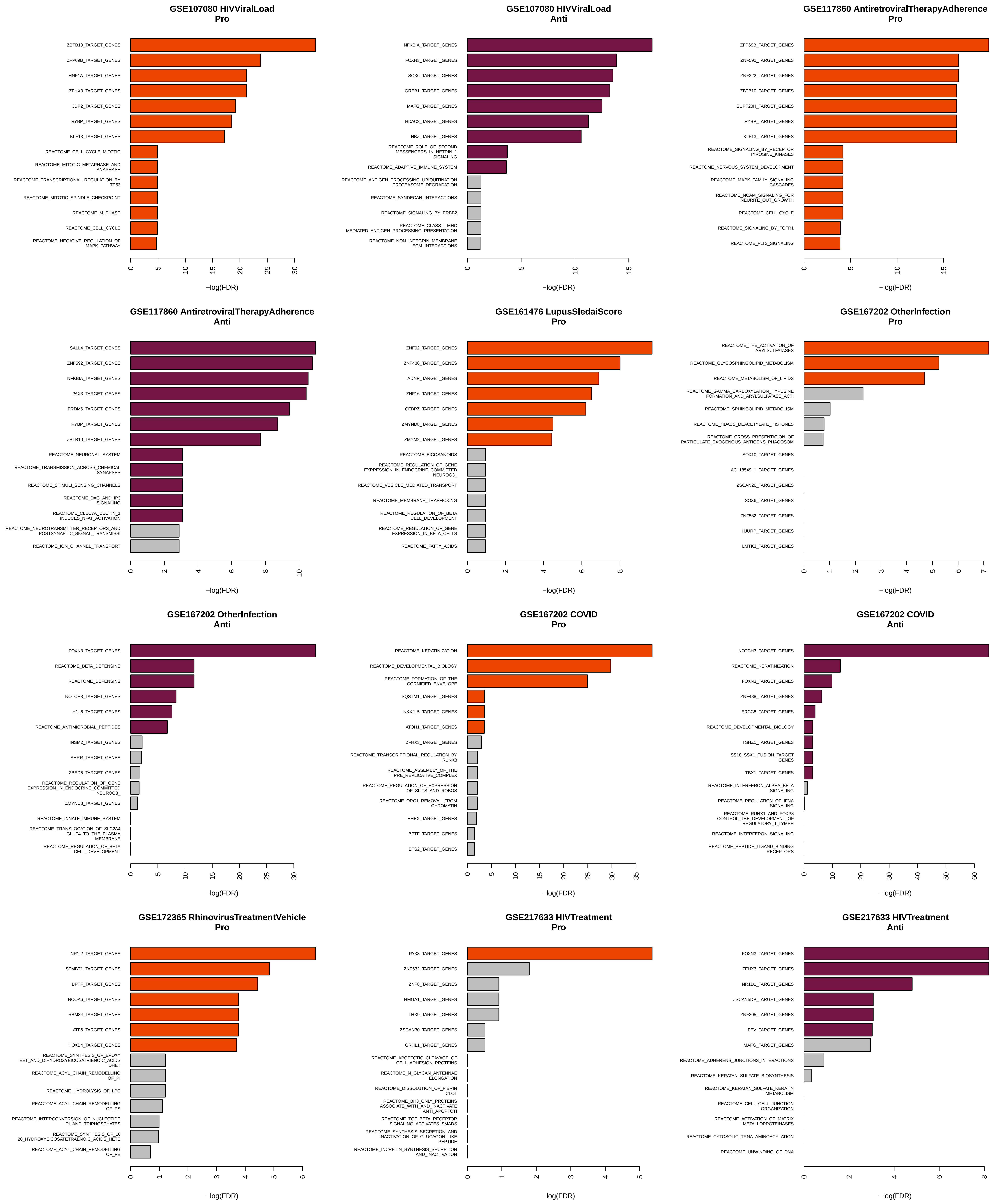
GSE107080 HIVViralLoad
Pro
ZBTB10_TARGET_GENES
ZFP69B_TARGET_GENES
HNF1A_TARGET_GENES
ZFHX3_TARGET_GENES
JDP2_TARGET_GENES
RYBP_TARGET_GENES
KLF13_TARGET_GENES
REACTOME_CELL_CYCLE_MITOTIC
REACTOME_MITOTIC_METAPHASE_AND ANAPHASE
REACTOME_TRANSCRIPTIONAL_REGULATION_BY TP53
REACTOME_MITOTIC_SPINDLE_CHECKPOINT
REACTOME_M_PHASE
REACTOME_CELL_CYCLE
REACTOME_NEGATIVE_REGULATION_OF MAPK_PATHWAY
0 5 10 15 20 25 30
−log(FDR)
GSE107080 HIVViralLoad
Anti
NFKBIA_TARGET_GENES
FOXN3_TARGET_GENES
SOX6_TARGET_GENES
GREB1_TARGET_GENES
MAFG_TARGET_GENES
HDAC3_TARGET_GENES
HBZ_TARGET_GENES
REACTOME_ROLE_OF_SECOND MESSENGERS_IN_NETRIN_1 SIGNALING
REACTOME_ADAPTIVE_IMMUNE_SYSTEM
REACTOME_ANTIGEN_PROCESSING_UBIQUITINATION PROTEASOME_DEGRADATION
REACTOME_SYNDECAN_INTERACTIONS
REACTOME_SIGNALING_BY_ERBB2
REACTOME_CLASS_I_MHC MEDIATED_ANTIGEN_PROCESSING_PRESENTATION
REACTOME_NON_INTEGRIN_MEMBRANE ECM_INTERACTIONS
0 5 10 15
−log(FDR)
GSE117860 AntiretroviralTherapyAdherence
Pro
ZFP69B_TARGET_GENES
ZNF592_TARGET_GENES
ZNF322_TARGET_GENES
ZBTB10_TARGET_GENES
SUPT20H_TARGET_GENES
RYBP_TARGET_GENES
KLF13_TARGET_GENES
REACTOME_SIGNALING_BY_RECEPTOR TYROSINE_KINASES
REACTOME_NERVOUS_SYSTEM_DEVELOPMENT
REACTOME_MAPK_FAMILY_SIGNALING CASCADES
REACTOME_NCAM_SIGNALING_FOR NEURITE_OUT_GROWTH
REACTOME_CELL_CYCLE
REACTOME_SIGNALING_BY_FGFR1
REACTOME_FLT3_SIGNALING
0 5 10 15
−log(FDR)
GSE117860 AntiretroviralTherapyAdherence
Anti
SALL4_TARGET_GENES
ZNF592_TARGET_GENES
NFKBIA_TARGET_GENES
PAX3_TARGET_GENES
PRDM6_TARGET_GENES
RYBP_TARGET_GENES
ZBTB10_TARGET_GENES
REACTOME_NEURONAL_SYSTEM
REACTOME_TRANSMISSION_ACROSS_CHEMICAL SYNAPSES
REACTOME_STIMULI_SENSING_CHANNELS
REACTOME_DAG_AND_IP3 SIGNALING
REACTOME_CLEC7A_DECTIN_1 INDUCES_NFAT_ACTIVATION
REACTOME_NEUROTRANSMITTER_RECEPTORS_AND POSTSYNAPTIC_SIGNAL_TRANSMISSI
REACTOME_ION_CHANNEL_TRANSPORT
0 2 4 6 8 10
−log(FDR)
GSE161476 LupusSledaiScore
Pro
ZNF92_TARGET_GENES
ZNF436_TARGET_GENES
ADNP_TARGET_GENES
ZNF16_TARGET_GENES
CEBPZ_TARGET_GENES
ZMYND8_TARGET_GENES
ZMYM2_TARGET_GENES
REACTOME_EICOSANOIDS
REACTOME_REGULATION_OF_GENE EXPRESSION_IN_ENDOCRINE_COMMITTED NEUROG3_
REACTOME_VESICLE_MEDIATED_TRANSPORT
REACTOME_MEMBRANE_TRAFFICKING
REACTOME_REGULATION_OF_BETA CELL_DEVELOPMENT
REACTOME_REGULATION_OF_GENE EXPRESSION_IN_BETA_CELLS
REACTOME_FATTY_ACIDS
0 2 4 6 8
−log(FDR)
GSE167202 OtherInfection
Pro
REACTOME_THE_ACTIVATION_OF ARYLSULFATASES
REACTOME_GLYCOSPHINGOLIPID_METABOLISM
REACTOME_METABOLISM_OF_LIPIDS
REACTOME_GAMMA_CARBOXYLATION_HYPUSINE FORMATION_AND_ARYLSULFATASE_ACTI
REACTOME_SPHINGOLIPID_METABOLISM
REACTOME_HDACS_DEACETYLATE_HISTONES
REACTOME_CROSS_PRESENTATION_OF PARTICULATE_EXOGENOUS_ANTIGENS_PHAGOSOM
SOX10_TARGET_GENES
AC118549_1_TARGET_GENES
ZSCAN26_TARGET_GENES
SOX6_TARGET_GENES
ZNF582_TARGET_GENES
HJURP_TARGET_GENES
LMTK3_TARGET_GENES
0 1 2 3 4 5 6 7
−log(FDR)
GSE167202 OtherInfection
Anti
FOXN3_TARGET_GENES
REACTOME_BETA_DEFENSINS
REACTOME_DEFENSINS
NOTCH3_TARGET_GENES
H1_6_TARGET_GENES
REACTOME_ANTIMICROBIAL_PEPTIDES
INSM2_TARGET_GENES
AHRR_TARGET_GENES
ZBED5_TARGET_GENES
REACTOME_REGULATION_OF_GENE EXPRESSION_IN_ENDOCRINE_COMMITTED NEUROG3_
ZMYND8_TARGET_GENES
REACTOME_INNATE_IMMUNE_SYSTEM
REACTOME_TRANSLOCATION_OF_SLC2A4 GLUT4_TO_THE_PLASMA MEMBRANE
REACTOME_REGULATION_OF_BETA CELL_DEVELOPMENT
0 5 10 15 20 25 30
−log(FDR)
GSE167202 COVID
Pro
REACTOME_KERATINIZATION
REACTOME_DEVELOPMENTAL_BIOLOGY
REACTOME_FORMATION_OF_THE CORNIFIED_ENVELOPE
SQSTM1_TARGET_GENES
NKX2_5_TARGET_GENES
ATOH1_TARGET_GENES
ZFHX3_TARGET_GENES
REACTOME_TRANSCRIPTIONAL_REGULATION_BY RUNX3
REACTOME_ASSEMBLY_OF_THE PRE_REPLICATIVE_COMPLEX
REACTOME_REGULATION_OF_EXPRESSION OF_SLITS_AND_ROBOS
REACTOME_ORC1_REMOVAL_FROM CHROMATIN
HHEX_TARGET_GENES
BPTF_TARGET_GENES
ETS2_TARGET_GENES
0 5 10 15 20 25 30 35
−log(FDR)
GSE167202 COVID
Anti
NOTCH3_TARGET_GENES
REACTOME_KERATINIZATION
FOXN3_TARGET_GENES
ZNF488_TARGET_GENES
ERCC8_TARGET_GENES
REACTOME_DEVELOPMENTAL_BIOLOGY
TSHZ1_TARGET_GENES
SS18_SSX1_FUSION_TARGET GENES
TBX1_TARGET_GENES
REACTOME_INTERFERON_ALPHA_BETA SIGNALING
REACTOME_REGULATION_OF_IFNA SIGNALING
REACTOME_RUNX1_AND_FOXP3 CONTROL_THE_DEVELOPMENT_OF REGULATORY_T_LYMPH
REACTOME_INTERFERON_SIGNALING
REACTOME_PEPTIDE_LIGAND_BINDING RECEPTORS
0 10 20 30 40 50 60
−log(FDR)
GSE172365 RhinovirusTreatmentVehicle
Pro
NR1I2_TARGET_GENES
SFMBT1_TARGET_GENES
BPTF_TARGET_GENES
NCOA6_TARGET_GENES
RBM34_TARGET_GENES
ATF6_TARGET_GENES
HOXB4_TARGET_GENES
REACTOME_SYNTHESIS_OF_EPOXY EET_AND_DIHYDROXYEICOSATRIENOIC_ACIDS DHET
REACTOME_ACYL_CHAIN_REMODELLING OF_PI
REACTOME_HYDROLYSIS_OF_LPC
REACTOME_ACYL_CHAIN_REMODELLING OF_PS
REACTOME_INTERCONVERSION_OF_NUCLEOTIDE DI_AND_TRIPHOSPHATES
REACTOME_SYNTHESIS_OF_16 20_HYDROXYEICOSATETRAENOIC_ACIDS_HETE
REACTOME_ACYL_CHAIN_REMODELLING OF_PE
0 1 2 3 4 5 6
−log(FDR)
GSE217633 HIVTreatment
Pro
PAX3_TARGET_GENES
ZNF532_TARGET_GENES
ZNF8_TARGET_GENES
HMGA1_TARGET_GENES
LHX9_TARGET_GENES
ZSCAN30_TARGET_GENES
GRHL1_TARGET_GENES
REACTOME_APOPTOTIC_CLEAVAGE_OF CELL_ADHESION_PROTEINS
REACTOME_N_GLYCAN_ANTENNAE ELONGATION
REACTOME_DISSOLUTION_OF_FIBRIN CLOT
REACTOME_BH3_ONLY_PROTEINS ASSOCIATE_WITH_AND_INACTIVATE ANTI_APOPTOTI
REACTOME_TGF_BETA_RECEPTOR SIGNALING_ACTIVATES_SMADS
REACTOME_SYNTHESIS_SECRETION_AND INACTIVATION_OF_GLUCAGON_LIKE PEPTIDE
REACTOME_INCRETIN_SYNTHESIS_SECRETION AND_INACTIVATION
0 1 2 3 4 5
−log(FDR)
GSE217633 HIVTreatment
Anti
FOXN3_TARGET_GENES
ZFHX3_TARGET_GENES
NR1D1_TARGET_GENES
ZSCAN5DP_TARGET_GENES
ZNF205_TARGET_GENES
FEV_TARGET_GENES
MAFG_TARGET_GENES
REACTOME_ADHERENS_JUNCTIONS_INTERACTIONS
REACTOME_KERATAN_SULFATE_BIOSYNTHESIS
REACTOME_KERATAN_SULFATE_KERATIN METABOLISM
REACTOME_CELL_CELL_JUNCTION ORGANIZATION
REACTOME_ACTIVATION_OF_MATRIX METALLOPROTEINASES
REACTOME_CYTOSOLIC_TRNA_AMINOACYLATION
REACTOME_UNWINDING_OF_DNA
0 2 4 6 8
−log(FDR)
