# Supplementary material for: Various diseases and conditions are strongly associated with the next-generation epigenetic aging clock CheekAge
Source: GeroScience. 2025 Mar 7;47(3):3191–206. doi: 10.1007/s11357-025-01579-9 (PMC12181163; doi:10.1007/s11357-025-01579-9)
Supplement: Supplementary file 3 — Supplementary Fig. 3 Enrichment plots for pulmonary and metabolic datasets. Transcription factor target and Reactome pathway enrichment results are shown for both “pro” and “anti” CpG groups that respectively promote or antagonize CheekAge’s ability to associate with signals in pulmonary and metabolic datasets. Significant results for “pro” CpGs are shown in orange while significant results for “anti” CpGs are shown in purple. Non-significant results are colored grey (PDF 12 KB) [file 11357_2025_1579_MOESM3_ESM.pdf]

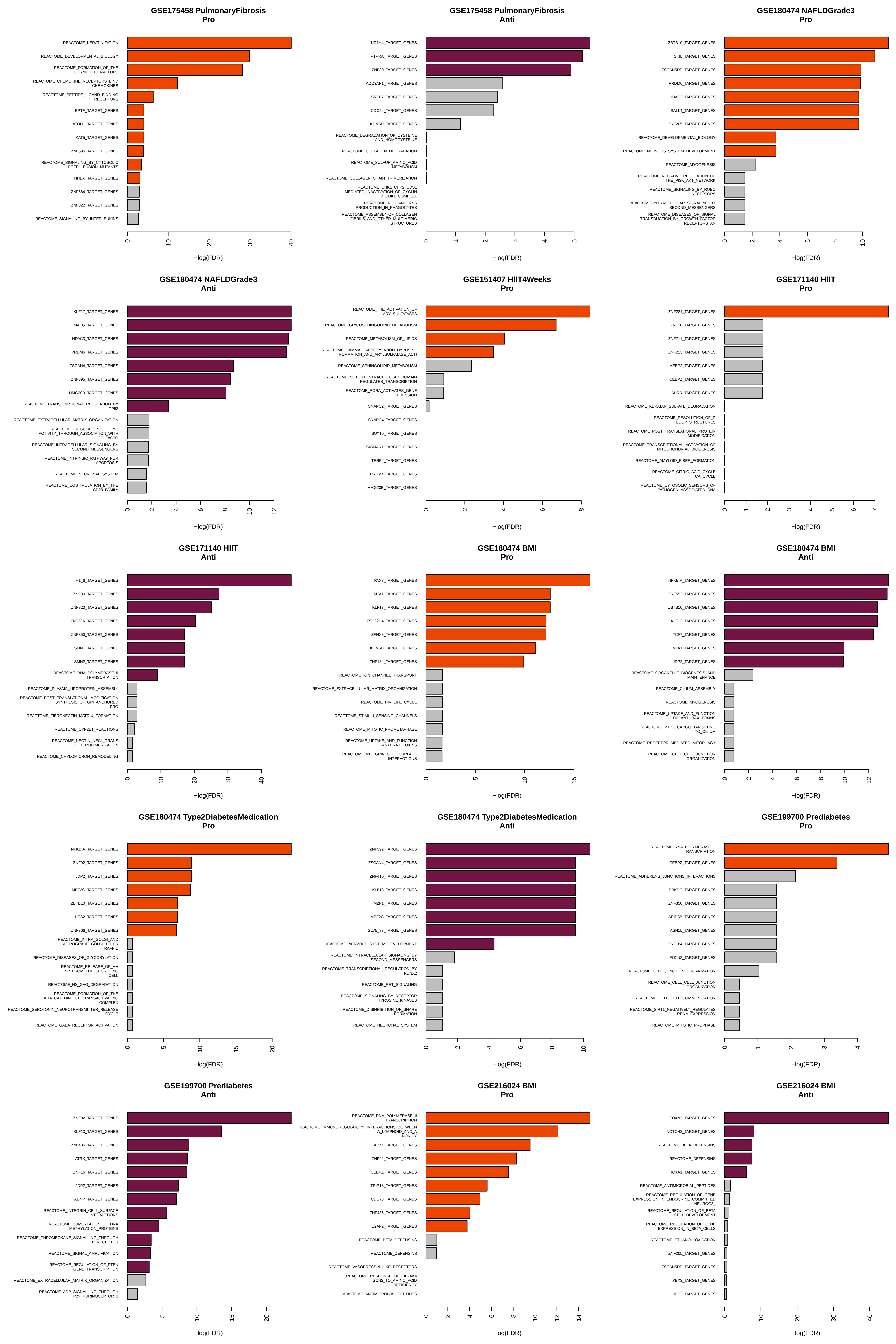
GSE175458 PulmonaryFibrosis
Pro
REACTOME_KERATINIZATION
REACTOME_DEVELOPMENTAL_BIOLOGY
REACTOME_FORMATION_OF_THE CORNIFIED_ENVELOPE
REACTOME_CHEMOKINE_RECEPTORS_BIND CHEMOKINES
REACTOME_PEPTIDE_LIGAND_BINDING RECEPTORS
BPTF_TARGET_GENES
ATOH1_TARGET_GENES
KAT5_TARGET_GENES
ZNF595_TARGET_GENES
REACTOME_SIGNALING_BY_CYTOSOLIC FGFR1_FUSION_MUTANTS
HHEX_TARGET_GENES
ZNF664_TARGET_GENES
ZNF322_TARGET_GENES
REACTOME_SIGNALING_BY_INTERLEUKINS
−log(FDR)
GSE175458 PulmonaryFibrosis
Anti
NR1H4_TARGET_GENES
PTPRA_TARGET_GENES
ZNF30_TARGET_GENES
ADCYAP1_TARGET_GENES
SRSF7_TARGET_GENES
CDC5L_TARGET_GENES
KDM5D_TARGET_GENES
REACTOME_DEGRADATION_OF_CYSTEINE AND_HOMOCYSTEINE
REACTOME_COLLAGEN_DEGRADATION
REACTOME_SULFUR_AMINO_ACID METABOLISM
REACTOME_COLLAGEN_CHAIN_TRIMERIZATION
REACTOME_CHK1_CHK2_CDS1 MEDIATED_INACTIVATION_OF_CYCLIN B_CDK1_COMPLEX
REACTOME_ROS_AND_RNS PRODUCTION_IN_PHAGOCYTES
REACTOME_ASSEMBLY_OF_COLLAGEN FIBRILS_AND_OTHER_MULTIMERIC STRUCTURES
−log(FDR)
GSE180474 NAFLDGrade3
Pro
ZBTB10_TARGET_GENES
SKIL_TARGET_GENES
ZSCAN5DP_TARGET_GENES
PRDM6_TARGET_GENES
HDAC3_TARGET_GENES
SALL4_TARGET_GENES
ZNF205_TARGET_GENES
REACTOME_DEVELOPMENTAL_BIOLOGY
REACTOME_NERVOUS_SYSTEM_DEVELOPMENT
REACTOME_MYOGENESIS
REACTOME_NEGATIVE_REGULATION_OF THE_PI3K_AKT_NETWORK
REACTOME_SIGNALING_BY_ROBO RECEPTORS
REACTOME_INTRACELLULAR_SIGNALING_BY SECOND_MESSENGERS
REACTOME_DISEASES_OF_SIGNAL TRANSDUCTION_BY_GROWTH_FACTOR RECEPTORS_AN
−log(FDR)
GSE180474 NAFLDGrade3
Anti
KLF17_TARGET_GENES
MAFG_TARGET_GENES
HDAC3_TARGET_GENES
PRDM6_TARGET_GENES
ZSCAN4_TARGET_GENES
ZNF395_TARGET_GENES
HMG20B_TARGET_GENES
REACTOME_TRANSCRIPTIONAL_REGULATION_BY TP53
REACTOME_EXTRACELLULAR_MATRIX_ORGANIZATION
REACTOME_REGULATION_OF_TP53 ACTIVITY_THROUGH_ASSOCIATION_WITH CO_FACTO
REACTOME_INTRACELLULAR_SIGNALING_BY SECOND_MESSENGERS
REACTOME_INTRINSIC_PATHWAY_FOR APOPTOSIS
REACTOME_NEURONAL_SYSTEM
REACTOME_COSTIMULATION_BY_THE CD28_FAMILY
−log(FDR)
GSE151407 HIIT4Weeks
Pro
REACTOME_THE_ACTIVATION_OF ARYLSULFATASES
REACTOME_GLYCOSPHINGOLIPID_METABOLISM
REACTOME_METABOLISM_OF_LIPIDS
REACTOME_GAMMA_CARBOXYLATION_HYPUSINE FORMATION_AND_ARYLSULFATASE_ACTI
REACTOME_SPHINGOLIPID_METABOLISM
REACTOME_NOTCH1_INTRACELLULAR_DOMAIN REGULATES_TRANSCRIPTION
REACTOME_RORA_ACTIVATES_GENE EXPRESSION
SNAPC2_TARGET_GENES
SNAPC4_TARGET_GENES
SOX10_TARGET_GENES
SIGMAR1_TARGET_GENES
TERF2_TARGET_GENES
PRDM4_TARGET_GENES
HMG20B_TARGET_GENES
−log(FDR)
GSE171140 HIIT
Pro
ZNF224_TARGET_GENES
ZNF10_TARGET_GENES
ZNF711_TARGET_GENES
ZNF213_TARGET_GENES
AEBP2_TARGET_GENES
CEBPZ_TARGET_GENES
AHRR_TARGET_GENES
REACTOME_KERATAN_SULFATE_DEGRADATION
REACTOME_RESOLUTION_OF_D LOOP_STRUCTURES
REACTOME_POST_TRANSLATIONAL_PROTEIN MODIFICATION
REACTOME_TRANSCRIPTIONAL_ACTIVATION_OF MITOCHONDRIAL_BIOGENESIS
REACTOME_AMYLOID_FIBER_FORMATION
REACTOME_CITRIC_ACID_CYCLE TCA_CYCLE
REACTOME_CYTOSOLIC_SENSORS_OF PATHOGEN_ASSOCIATED_DNA
−log(FDR)
GSE171140 HIIT
Anti
H1_6_TARGET_GENES
ZNF30_TARGET_GENES
ZNF320_TARGET_GENES
ZNF33A_TARGET_GENES
ZNF350_TARGET_GENES
SMN1_TARGET_GENES
SMN2_TARGET_GENES
REACTOME_RNA_POLYMERASE_II TRANSCRIPTION
REACTOME_PLASMA_LIPOPROTEIN_ASSEMBLY
REACTOME_POST_TRANSLATIONAL_MODIFICATION SYNTHESIS_OF_GPI_ANCHORED PRO
REACTOME_FIBRONECTIN_MATRIX_FORMATION
REACTOME_CYP2E1_REACTIONS
REACTOME_NECTIN_NECL_TRANS HETERODIMERIZATION
REACTOME_CHYLOMICRON_REMODELING
−log(FDR)
GSE180474 BMI
Pro
PAX3_TARGET_GENES
MTA1_TARGET_GENES
KLF17_TARGET_GENES
TSC22D4_TARGET_GENES
ZFHX3_TARGET_GENES
KDM5D_TARGET_GENES
ZNF184_TARGET_GENES
REACTOME_ION_CHANNEL_TRANSPORT
REACTOME_EXTRACELLULAR_MATRIX_ORGANIZATION
REACTOME_HIV_LIFE_CYCLE
REACTOME_STIMULI_SENSING_CHANNELS
REACTOME_MITOTIC_PROMETAPHASE
REACTOME_UPTAKE_AND_FUNCTION OF_ANTHRAX_TOXINS
REACTOME_INTEGRIN_CELL_SURFACE INTERACTIONS
−log(FDR)
GSE180474 BMI
Anti
NFKBIA_TARGET_GENES
ZNF592_TARGET_GENES
ZBTB10_TARGET_GENES
KLF13_TARGET_GENES
TCF7_TARGET_GENES
MTA1_TARGET_GENES
JDP2_TARGET_GENES
REACTOME_ORGANELLE_BIOGENESIS_AND MAINTENANCE
REACTOME_CILIUM_ASSEMBLY
REACTOME_MYOGENESIS
REACTOME_UPTAKE_AND_FUNCTION OF_ANTHRAX_TOXINS
REACTOME_VXPX_CARGO_TARGETING TO_CILIUM
REACTOME_RECEPTOR_MEDIATED_MITOPHAGY
REACTOME_CELL_CELL_JUNCTION ORGANIZATION
−log(FDR)
GSE180474 Type2DiabetesMedication
Pro
NFKBIA_TARGET_GENES
ZNF92_TARGET_GENES
JDP2_TARGET_GENES
MEF2C_TARGET_GENES
ZBTB10_TARGET_GENES
HES2_TARGET_GENES
ZNF766_TARGET_GENES
REACTOME_INTRA_GOLGI_AND RETROGRADE_GOLGI_TO_ER TRAFFIC
REACTOME_DISEASES_OF_GLYCOSYLATION
REACTOME_RELEASE_OF_HH NP_FROM_THE_SECRETING CELL
REACTOME_HS_GAG_DEGRADATION
REACTOME_FORMATION_OF_THE BETA_CATENIN_TCF_TRANSACTIVATING COMPLEX
REACTOME_SEROTONIN_NEUROTRANSMITTER_RELEASE CYCLE
REACTOME_GABA_RECEPTOR_ACTIVATION
−log(FDR)
GSE180474 Type2DiabetesMedication
Anti
ZNF592_TARGET_GENES
ZSCAN4_TARGET_GENES
ZNF410_TARGET_GENES
KLF13_TARGET_GENES
MZF1_TARGET_GENES
MEF2C_TARGET_GENES
IGLV5_37_TARGET_GENES
REACTOME_NERVOUS_SYSTEM_DEVELOPMENT
REACTOME_INTRACELLULAR_SIGNALING_BY SECOND_MESSENGERS
REACTOME_TRANSCRIPTIONAL_REGULATION_BY RUNX2
REACTOME_RET_SIGNALING
REACTOME_SIGNALING_BY_RECEPTOR TYROSINE_KINASES
REACTOME_DISINHIBITION_OF_SNARE FORMATION
REACTOME_NEURONAL_SYSTEM
−log(FDR)
GSE199700 Prediabetes
Pro
REACTOME_RNA_POLYMERASE_II TRANSCRIPTION
CEBPZ_TARGET_GENES
REACTOME_ADHERENS_JUNCTIONS_INTERACTIONS
PRKDC_TARGET_GENES
ZNF350_TARGET_GENES
ARID3B_TARGET_GENES
ASH1L_TARGET_GENES
ZNF184_TARGET_GENES
FOXN3_TARGET_GENES
REACTOME_CELL_JUNCTION_ORGANIZATION
REACTOME_CELL_CELL_JUNCTION ORGANIZATION
REACTOME_CELL_CELL_COMMUNICATION
REACTOME_SIRT1_NEGATIVELY_REGULATES RRNA_EXPRESSION
REACTOME_MITOTIC_PROPHASE
−log(FDR)
GSE199700 Prediabetes
Anti
ZNF92_TARGET_GENES
KLF13_TARGET_GENES
ZNF436_TARGET_GENES
ATRX_TARGET_GENES
ZNF16_TARGET_GENES
JDP2_TARGET_GENES
ADNP_TARGET_GENES
REACTOME_INTEGRIN_CELL_SURFACE INTERACTIONS
REACTOME_SUMOYLATION_OF_DNA METHYLATION_PROTEINS
REACTOME_THROMBOXANE_SIGNALLING_THROUGH TP_RECEPTOR
REACTOME_SIGNAL_AMPLIFICATION
REACTOME_REGULATION_OF_PTEN GENE_TRANSCRIPTION
REACTOME_EXTRACELLULAR_MATRIX_ORGANIZATION
REACTOME_ADP_SIGNALLING_THROUGH P2Y_PURINOCEPTOR_1
−log(FDR)
GSE216024 BMI
Pro
REACTOME_RNA_POLYMERASE_II TRANSCRIPTION
REACTOME_IMMUNOREGULATORY_INTERACTIONS_BETWEEN A_LYMPHOID_AND_A NON_LY
ATRX_TARGET_GENES
ZNF92_TARGET_GENES
CEBPZ_TARGET_GENES
TRIP13_TARGET_GENES
CDC73_TARGET_GENES
ZNF436_TARGET_GENES
U2AF2_TARGET_GENES
REACTOME_BETA_DEFENSINS
REACTOME_DEFENSINS
REACTOME_VASOPRESSIN_LIKE_RECEPTORS
REACTOME_RESPONSE_OF_EIF2AK4 GCN2_TO_AMINO_ACID DEFICIENCY
REACTOME_ANTIMICROBIAL_PEPTIDES
−log(FDR)
GSE216024 BMI
Anti
FOXN3_TARGET_GENES
NOTCH3_TARGET_GENES
REACTOME_BETA_DEFENSINS
REACTOME_DEFENSINS
HOXA1_TARGET_GENES
REACTOME_ANTIMICROBIAL_PEPTIDES
REACTOME_REGULATION_OF_GENE EXPRESSION_IN_ENDOCRINE_COMMITTED NEUROG3_
REACTOME_REGULATION_OF_BETA CELL_DEVELOPMENT
REACTOME_REGULATION_OF_GENE EXPRESSION_IN_BETA_CELLS
REACTOME_ETHANOL_OXIDATION
ZNF205_TARGET_GENES
ZSCAN5DP_TARGET_GENES
YBX3_TARGET_GENES
JDP2_TARGET_GENES
−log(FDR)
