# Supplementary material for: Various diseases and conditions are strongly associated with the next-generation epigenetic aging clock CheekAge
Source: GeroScience. 2025 Mar 7;47(3):3191–206. doi: 10.1007/s11357-025-01579-9 (PMC12181163; doi:10.1007/s11357-025-01579-9)
Supplement: Supplementary file 4 — Supplementary Fig. 4 Enrichment plots for aging, psychological, drug, treatment, and exposure datasets. Transcription factor target and Reactome pathway enrichment results are shown for both “pro” and “anti” CpG groups that respectively promote or antagonize CheekAge’s ability to associate with signals in aging, psychological, drug, treatment, and exposure datasets. Significant results for “pro” CpGs are shown in orange while significant results for “anti” CpGs are shown in purple. Non-significant results are colored grey (PDF 12 KB) [file 11357_2025_1579_MOESM4_ESM.pdf]

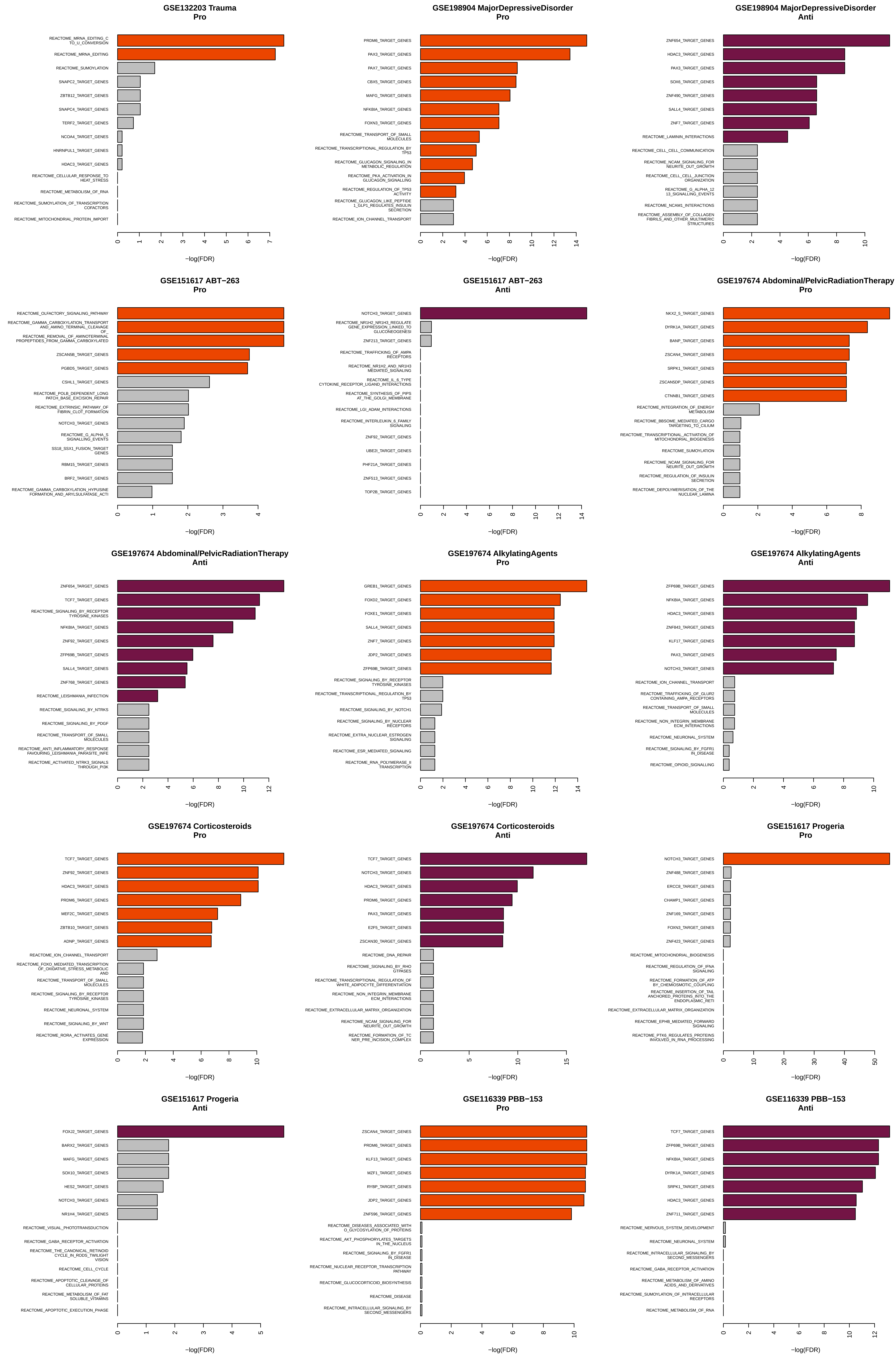
GSE132203 Trauma
Pro
REACTOME_MRNA_EDITING_C TO_U_CONVERSION
REACTOME_MRNA_EDITING
REACTOME_SUMOYLATION
SNAPC2_TARGET_GENES
ZBTB12_TARGET_GENES
SNAPC4_TARGET_GENES
TERF2_TARGET_GENES
NCOA4_TARGET_GENES
HNRNPUL1_TARGET_GENES
HDAC3_TARGET_GENES
REACTOME_CELLULAR_RESPONSE_TO HEAT_STRESS
REACTOME_METABOLISM_OF_RNA
REACTOME_SUMOYLATION_OF_TRANSCRIPTION COFACTORS
REACTOME_MITOCHONDRIAL_PROTEIN_IMPORT
−log(FDR)
GSE198904 MajorDepressiveDisorder
Pro
PRDM6_TARGET_GENES
PAX3_TARGET_GENES
PAX7_TARGET_GENES
CBX5_TARGET_GENES
MAFG_TARGET_GENES
NFKBIA_TARGET_GENES
FOXN3_TARGET_GENES
REACTOME_TRANSPORT_OF_SMALL MOLECULES
REACTOME_TRANSCRIPTIONAL_REGULATION_BY TP53
REACTOME_GLUCAGON_SIGNALING_IN METABOLIC_REGULATION
REACTOME_PKA_ACTIVATION_IN GLUCAGON_SIGNALLING
REACTOME_REGULATION_OF_TP53 ACTIVITY
REACTOME_GLUCAGON_LIKE_PEPTIDE 1_GLP1_REGULATES_INSULIN SECRETION
REACTOME_ION_CHANNEL_TRANSPORT
−log(FDR)
GSE198904 MajorDepressiveDisorder
Anti
ZNF654_TARGET_GENES
HDAC3_TARGET_GENES
PAX3_TARGET_GENES
SOX6_TARGET_GENES
ZNF490_TARGET_GENES
SALL4_TARGET_GENES
ZNF7_TARGET_GENES
REACTOME_LAMININ_INTERACTIONS
REACTOME_CELL_CELL_COMMUNICATION
REACTOME_NCAM_SIGNALING_FOR NEURITE_OUT_GROWTH
REACTOME_CELL_CELL_JUNCTION ORGANIZATION
REACTOME_G_ALPHA_12 13_SIGNALLING_EVENTS
REACTOME_NCAM1_INTERACTIONS
REACTOME_ASSEMBLY_OF_COLLAGEN FIBRILS_AND_OTHER_MULTIMERIC STRUCTURES
−log(FDR)
GSE151617 ABT−263
Pro
REACTOME_OLFACTORY_SIGNALING_PATHWAY
REACTOME_GAMMA_CARBOXYLATION_TRANSPORT AND_AMINO_TERMINAL_CLEAVAGE OF_
REACTOME_REMOVAL_OF_AMINOTERMINAL PROPEPTIDES_FROM_GAMMA_CARBOXYLATED
ZSCAN5B_TARGET_GENES
PGBD5_TARGET_GENES
CSHL1_TARGET_GENES
REACTOME_POLB_DEPENDENT_LONG PATCH_BASE_EXCISION_REPAIR
REACTOME_EXTRINSIC_PATHWAY_OF FIBRIN_CLOT_FORMATION
NOTCH3_TARGET_GENES
REACTOME_G_ALPHA_S SIGNALLING_EVENTS
SS18_SSX1_FUSION_TARGET GENES
RBM15_TARGET_GENES
BRF2_TARGET_GENES
REACTOME_GAMMA_CARBOXYLATION_HYPUSINE FORMATION_AND_ARYLSULFATASE_ACTI
−log(FDR)
GSE151617 ABT−263
Anti
NOTCH3_TARGET_GENES
REACTOME_NR1H2_NR1H3_REGULATE GENE_EXPRESSION_LINKED_TO GLUCONEOGENESI
ZNF213_TARGET_GENES
REACTOME_TRAFFICKING_OF_AMPA RECEPTORS
REACTOME_NR1H2_AND_NR1H3 MEDIATED_SIGNALING
REACTOME_IL_6_TYPE CYTOKINE_RECEPTOR_LIGAND_INTERACTIONS
REACTOME_SYNTHESIS_OF_PIPS AT_THE_GOLGI_MEMBRANE
REACTOME_LGI_ADAM_INTERACTIONS
REACTOME_INTERLEUKIN_6_FAMILY SIGNALING
ZNF92_TARGET_GENES
UBE2I_TARGET_GENES
PHF21A_TARGET_GENES
ZNF513_TARGET_GENES
TOP2B_TARGET_GENES
−log(FDR)
GSE197674 Abdominal/PelvicRadiationTherapy
Pro
NKX2_5_TARGET_GENES
DYRK1A_TARGET_GENES
BANP_TARGET_GENES
ZSCAN4_TARGET_GENES
SRPK1_TARGET_GENES
ZSCAN5DP_TARGET_GENES
CTNNB1_TARGET_GENES
REACTOME_INTEGRATION_OF_ENERGY METABOLISM
REACTOME_BBSOME_MEDIATED_CARGO TARGETING_TO_CILIUM
REACTOME_TRANSCRIPTIONAL_ACTIVATION_OF MITOCHONDRIAL_BIOGENESIS
REACTOME_SUMOYLATION
REACTOME_NCAM_SIGNALING_FOR NEURITE_OUT_GROWTH
REACTOME_REGULATION_OF_INSULIN SECRETION
REACTOME_DEPOLYMERISATION_OF_THE NUCLEAR_LAMINA
−log(FDR)
GSE197674 Abdominal/PelvicRadiationTherapy
Anti
ZNF654_TARGET_GENES
TCF7_TARGET_GENES
REACTOME_SIGNALING_BY_RECEPTOR TYROSINE_KINASES
NFKBIA_TARGET_GENES
ZNF92_TARGET_GENES
ZFP69B_TARGET_GENES
SALL4_TARGET_GENES
ZNF768_TARGET_GENES
REACTOME_LEISHMANIA_INFECTION
REACTOME_SIGNALING_BY_NTRKS
REACTOME_SIGNALING_BY_PDGF
REACTOME_TRANSPORT_OF_SMALL MOLECULES
REACTOME_ANTI_INFLAMMATORY_RESPONSE FAVOURING_LEISHMANIA_PARASITE_INFE
REACTOME_ACTIVATED_NTRK3_SIGNALS THROUGH_PI3K
−log(FDR)
GSE197674 AlkylatingAgents
Pro
GREB1_TARGET_GENES
FOXD2_TARGET_GENES
FOXE1_TARGET_GENES
SALL4_TARGET_GENES
ZNF7_TARGET_GENES
JDP2_TARGET_GENES
ZFP69B_TARGET_GENES
REACTOME_SIGNALING_BY_RECEPTOR TYROSINE_KINASES
REACTOME_TRANSCRIPTIONAL_REGULATION_BY TP53
REACTOME_SIGNALING_BY_NOTCH1
REACTOME_SIGNALING_BY_NUCLEAR RECEPTORS
REACTOME_EXTRA_NUCLEAR_ESTROGEN SIGNALING
REACTOME_ESR_MEDIATED_SIGNALING
REACTOME_RNA_POLYMERASE_II TRANSCRIPTION
−log(FDR)
GSE197674 AlkylatingAgents
Anti
ZFP69B_TARGET_GENES
NFKBIA_TARGET_GENES
HDAC3_TARGET_GENES
ZNF843_TARGET_GENES
KLF17_TARGET_GENES
PAX3_TARGET_GENES
NOTCH3_TARGET_GENES
REACTOME_ION_CHANNEL_TRANSPORT
REACTOME_TRAFFICKING_OF_GLUR2 CONTAINING_AMPA_RECEPTORS
REACTOME_TRANSPORT_OF_SMALL MOLECULES
REACTOME_NON_INTEGRIN_MEMBRANE ECM_INTERACTIONS
REACTOME_NEURONAL_SYSTEM
REACTOME_SIGNALING_BY_FGFR1 IN_DISEASE
REACTOME_OPIOID_SIGNALLING
−log(FDR)
GSE197674 Corticosteroids
Pro
TCF7_TARGET_GENES
ZNF92_TARGET_GENES
HDAC3_TARGET_GENES
PRDM6_TARGET_GENES
MEF2C_TARGET_GENES
ZBTB10_TARGET_GENES
ADNP_TARGET_GENES
REACTOME_ION_CHANNEL_TRANSPORT
REACTOME_FOXO_MEDIATED_TRANSCRIPTION OF_OXIDATIVE_STRESS_METABOLIC AND
REACTOME_TRANSPORT_OF_SMALL MOLECULES
REACTOME_SIGNALING_BY_RECEPTOR TYROSINE_KINASES
REACTOME_NEURONAL_SYSTEM
REACTOME_SIGNALING_BY_WNT
REACTOME_RORA_ACTIVATES_GENE EXPRESSION
−log(FDR)
GSE197674 Corticosteroids
Anti
TCF7_TARGET_GENES
NOTCH3_TARGET_GENES
HDAC3_TARGET_GENES
PRDM6_TARGET_GENES
PAX3_TARGET_GENES
E2F5_TARGET_GENES
ZSCAN30_TARGET_GENES
REACTOME_DNA_REPAIR
REACTOME_SIGNALING_BY_RHO GTPASES
REACTOME_TRANSCRIPTIONAL_REGULATION_OF WHITE_ADIPOCYTE_DIFFERENTIATION
REACTOME_NON_INTEGRIN_MEMBRANE ECM_INTERACTIONS
REACTOME_EXTRACELLULAR_MATRIX_ORGANIZATION
REACTOME_NCAM_SIGNALING_FOR NEURITE_OUT_GROWTH
REACTOME_FORMATION_OF_TC NER_PRE_INCISION_COMPLEX
−log(FDR)
GSE151617 Progeria
Pro
NOTCH3_TARGET_GENES
ZNF488_TARGET_GENES
ERCC8_TARGET_GENES
CHAMP1_TARGET_GENES
ZNF169_TARGET_GENES
FOXN3_TARGET_GENES
ZNF423_TARGET_GENES
REACTOME_MITOCHONDRIAL_BIOGENESIS
REACTOME_REGULATION_OF_IFNA SIGNALING
REACTOME_FORMATION_OF_ATP BY_CHEMIOSMOTIC_COUPLING
REACTOME_INSERTION_OF_TAIL ANCHORED_PROTEINS_INTO_THE ENDOPLASMIC_RETI
REACTOME_EXTRACELLULAR_MATRIX_ORGANIZATION
REACTOME_EPHB_MEDIATED_FORWARD SIGNALING
REACTOME_PTK6_REGULATES_PROTEINS INVOLVED_IN_RNA_PROCESSING
−log(FDR)
GSE151617 Progeria
Anti
FOXJ2_TARGET_GENES
BARX2_TARGET_GENES
MAFG_TARGET_GENES
SOX10_TARGET_GENES
HES2_TARGET_GENES
NOTCH3_TARGET_GENES
NR1H4_TARGET_GENES
REACTOME_VISUAL_PHOTOTRANSDUCTION
REACTOME_GABA_RECEPTOR_ACTIVATION
REACTOME_THE_CANONICAL_RETINOID CYCLE_IN_RODS_TWILIGHT VISION
REACTOME_CELL_CYCLE
REACTOME_APOPTOTIC_CLEAVAGE_OF CELLULAR_PROTEINS
REACTOME_METABOLISM_OF_FAT SOLUBLE_VITAMINS
REACTOME_APOPTOTIC_EXECUTION_PHASE
−log(FDR)
GSE116339 PBB−153
Pro
ZSCAN4_TARGET_GENES
PRDM6_TARGET_GENES
KLF13_TARGET_GENES
MZF1_TARGET_GENES
RYBP_TARGET_GENES
JDP2_TARGET_GENES
ZNF596_TARGET_GENES
REACTOME_DISEASES_ASSOCIATED_WITH O_GLYCOSYLATION_OF_PROTEINS
REACTOME_AKT_PHOSPHORYLATES_TARGETS IN_THE_NUCLEUS
REACTOME_SIGNALING_BY_FGFR1 IN_DISEASE
REACTOME_NUCLEAR_RECEPTOR_TRANSCRIPTION PATHWAY
REACTOME_GLUCOCORTICOID_BIOSYNTHESIS
REACTOME_DISEASE
REACTOME_INTRACELLULAR_SIGNALING_BY SECOND_MESSENGERS
−log(FDR)
GSE116339 PBB−153
Anti
TCF7_TARGET_GENES
ZFP69B_TARGET_GENES
NFKBIA_TARGET_GENES
DYRK1A_TARGET_GENES
SRPK1_TARGET_GENES
HDAC3_TARGET_GENES
ZNF711_TARGET_GENES
REACTOME_NERVOUS_SYSTEM_DEVELOPMENT
REACTOME_NEURONAL_SYSTEM
REACTOME_INTRACELLULAR_SIGNALING_BY SECOND_MESSENGERS
REACTOME_GABA_RECEPTOR_ACTIVATION
REACTOME_METABOLISM_OF_AMINO ACIDS_AND_DERIVATIVES
REACTOME_SUMOYLATION_OF_INTRACELLULAR RECEPTORS
REACTOME_METABOLISM_OF_RNA
−log(FDR)
