# Supplementary figures and images for: Various diseases and conditions are strongly associated with the next-generation epigenetic aging clock CheekAge
Source: GeroScience. 2025 Mar 7;47(3):3191–206. doi: 10.1007/s11357-025-01579-9 (PMC12181163; doi:10.1007/s11357-025-01579-9)

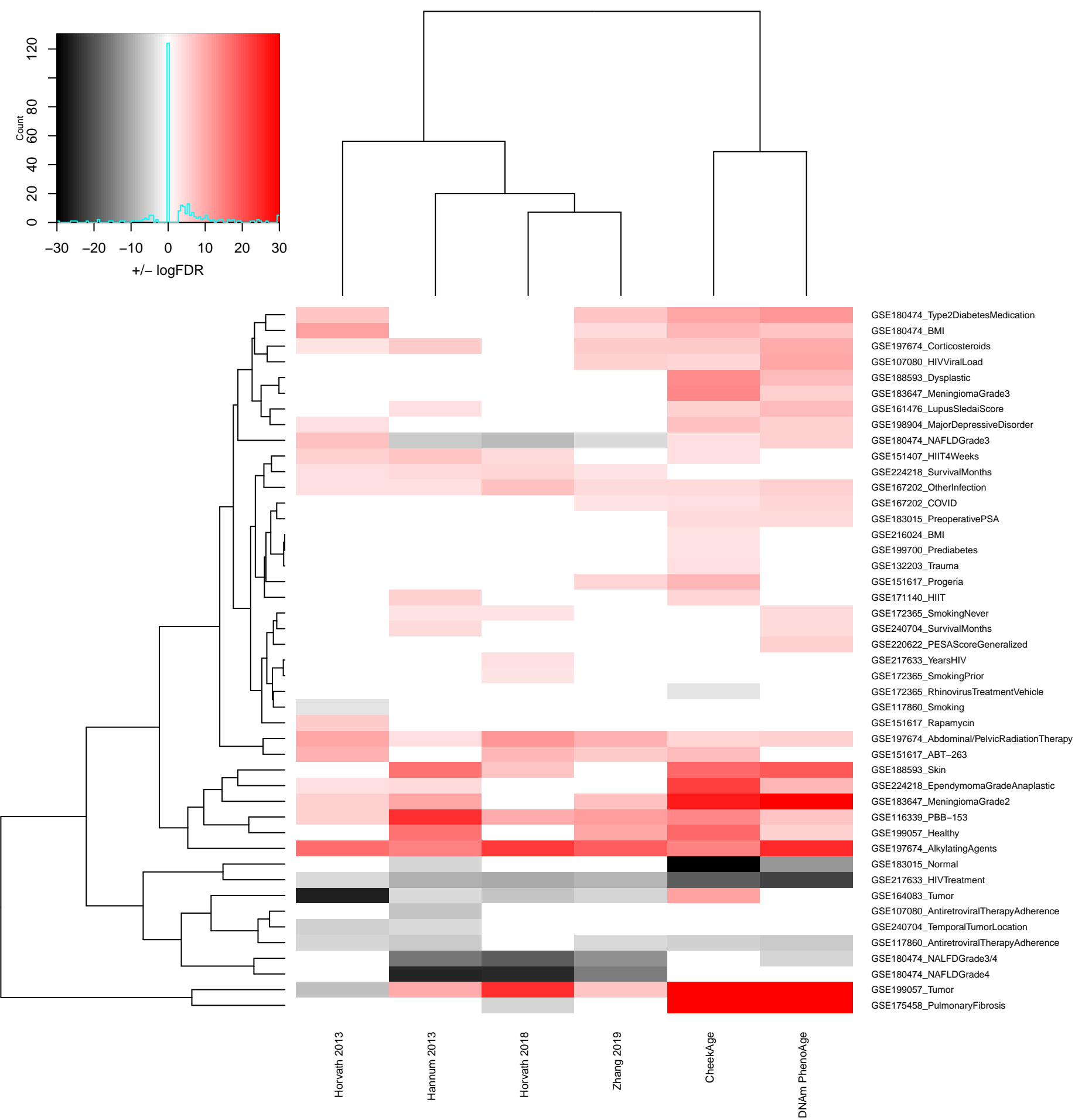

Supplement: Supplementary file 5 — Supplementary Fig. 5 Summary of association results for all clocks and datasets. The ability of each clock to associate with distinct signals is visualized. The color white indicates no significant association, the colors pink/red indicate a significant increase in delta age (epigenetic age – chronological age), and the colors grey/black indicate a significant decrease in delta age. Darker colors (red/black) indicate more significant associations than lighter colors (pink/grey) (PDF 36 KB) [file 11357_2025_1579_MOESM5_ESM.pdf]
